# Age is not just a number—Mathematical model suggests senescence affects how fish populations respond to different fishing regimes

**DOI:** 10.1002/ece3.8058

**Published:** 2021-09-07

**Authors:** Pauliina A. Ahti, Silva Uusi‐Heikkilä, Timo J. Marjomäki, Anna Kuparinen

**Affiliations:** ^1^ Department of Biological and Environmental Science University of Jyväskylä Jyväskylä Finland; ^2^ Institute of Biodiversity, Animal Health, and Comparative Medicine College of Medical, Veterinary, and Life Sciences University of Glasgow Glasgow UK

**Keywords:** eco‐evolutionary dynamics, fisheries, life‐history, senescence, trade‐offs

## Abstract

Senescence is often described as an age‐dependent increase in natural mortality (known as actuarial senescence) and an age‐dependent decrease in fecundity (known as reproductive senescence), and its role in nature is still poorly understood. Based on empirical estimates of reproductive and actuarial senescence, we used mathematical simulations to explore how senescence affects the population dynamics of *Coregonus albula*, a small, schooling salmonid fish. Using an empirically based eco‐evolutionary model, we investigated how the presence or absence of senescence affects the eco‐evolutionary dynamics of a fish population during pristine, intensive harvest, and recovery phases. Our simulation results showed that the presence or absence of senescence affected how the population responded to the selection regime. At an individual level, gillnetting caused a larger decline in asymptotic length when senescence was present, compared to the nonsenescent population, and the opposite occurred when fishing was done by trawling. This change was accompanied by evolution toward younger age at maturity. At the population level, the change in biomass and number of fish in response to different fishery size‐selection patterns depended on the presence or absence of senescence. Since most life‐history and fisheries models ignore senescence, they may be over‐estimating reproductive capacity and under‐estimating natural mortality. Our results highlight the need to understand the combined effects of life‐history characters such as senescence and fisheries selection regime to ensure the successful management of our natural resources.

## Graphical abstract

Empirical data from *Coregonus albula* were used to simulate senescence in fish population. The presences or absence of senescence affect the magnitude of fish evolutionary response to fishing and may affect how well the population recovers from fishing.

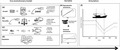

## 1 INTRODUCTION

Senescence is considered a fundamentally fitness decreasing trait, and its presence and role in natural populations remains an unsolved problem in biology (Monaghan et al., 2008; Nussey et al., 2013; Selman et al., 2012). Senescence is often described as age‐dependent increase in natural mortality (known as actuarial senescence) and age‐dependent decrease in reproductive function (known as reproductive senescence). For much of the 20th century, it was thought that very few animals in the wild experience senescence because external factors such as predators, diseases, or environmental stressors would kill them before the consequences of aging would commence (Medawar, 1952). Today, evidence for senescence across taxa is accumulating (Nussey et al., 2013). Several studies of wild populations have shown that trade‐offs exist between early‐ and late‐life performance (Bonsall & Mangel, 2004; Jensen, 1996; Lemaitre et al., 2014; Maklakov & Chapman, 2019), likely contributing to the onset or development of senescence in an individual. The early‐ versus late‐life performance has been tested in many vertebrates and has gained a lot of support, but tests in fishes are scarce, mainly owing to the difficulty of testing for senescence in species with indeterminate growth (Heino & Kaitala, 1999; Lemaître et al., 2015; Reznick et al., 2002).

Life‐history trade‐offs may take place between functions as well as within the same function. For instance, increased growth rate and increased reproductive effort early in life and higher natural mortality rate later in life are known to be linked (Kirkwood & Rose, 1991; Lester et al., 2004). Similarly, investment in future reproductive effort is thought to be of lesser value in terms of fitness benefits than current reproductive effort, mainly due to the uncertainty of future reproduction (Zhang & Hood, 2016). Following the close link between life‐history characters and senescence, it is therefore likely, that increased allocation to reproduction in early life leads to an increased rate of aging later as hypothesized by the antagonistic pleiotropy (Williams, 1957) and disposable soma theories (Kirkwood, 1977). As a consequence, fishing‐induced changes in maturation age and senescence could actually be linked (Benoît et al., 2018). Indeed, the natural mortality rate of many fish populations is thought to have increased in the recent decades (Gislason et al., 2010).

Given that most fishes express indeterminate growth and high longevity (Carey & Judge, 2000), it has been suggested that fish experience delayed senescence relative to birds and mammals, facilitated in part by the capacity for increasing fecundity with age (Reznick et al., 2002). Other works suggest they may completely lack senescent deterioration (Sauer et al., 2021; Vaupel et al., 2004). Indeed, female body size and reproductive output in fish are known to be positively correlated in many species, indicating that the older and larger the fish, the higher its reproductive output. Most fisheries and fish population models (Andersen & Beyer, 2015; Beverton & Holt, 1957; Enberg et al., 2010; Zimmermann & Jørgensen, 2015) and life‐history models (Brunel et al., 2013; Charnov et al., 2013; Roff, 1983) assume that body size (weight) scales isometrically with reproductive output. Recent meta‐analysis has provided cues that the scaling might even be hyperallometric (Barneche et al., 2018), further stressing the role of large and old individuals for population growth or, in case of over‐exploitation, for recovery. Additionally, in fisheries and life‐history models, the natural mortality of fish is often assumed to be independent of the age or size of the fish (Gislason et al., 2010). Models with increasing fecundity with age and size, and age‐ and size‐independent natural mortality essentially describe fish as having no reproductive or actuarial senescence at all.

While rarely included in fisheries and life‐history models, senescence in fish was first documented over 60 years ago (Comfort, 1960, 1963; Gerking, 1957; Woodhead & Ellett, 1966, 1967, 1969a, 1969b). Over the past few decades, evidence for both reproductive (Benoît et al., 2018; Reznick et al., 2006; Žák & Reichard, 2020) and actuarial (Beverton et al., 2004; Uriarte et al., 2016) senescence as well as general deterioration with age (Carlson et al., 2007; Hendry et al., 2004; Morbey et al., 2005; Patnaik et al., 1994) in fish has started to accumulate. While the importance of old and large individuals for the reproductive pool is evident, the common conservation measure of relying on the reproductive effort of large individuals could have detrimental effects on the recovery and resilience of fish populations if actuarial or reproductive senescence is indeed wide‐spread in fishes (Le Bris et al., 2015). Given that senescence influences the reproductive outcome and natural mortality rate, it would also have major consequences to our understanding of fish life‐histories and population dynamics.

Sustainable fish populations are vital not only for food security around the world (Merino et al., 2012), but also for healthy biodiversity and climate regulation (Jackson, 2008). The traditional density‐dependent population growth theory suggests that at low abundance populations should grow at a fast rate. Following this, fish stocks should recover quickly after fishing has been ceased. Yet, despite large‐scale fishing moratoriums, many fish stocks have not fully recovered from intense fishing and remain low (Bailey, 2011; Myers & Barrowman, 1997; Pedersen et al., 2017; Rougier et al., 2012), or are recovering at a lower rate than expected (Hutchings & Reynolds, 2004). The reasons behind the lack of recovery are complex and likely include factors such as habitat destruction, climatic conditions, trends in prey–predator relationships, and changes in life‐history traits (Dulvy et al., 2003; Hutchings & Reynolds, 2004; Olsen et al., 2009). While much research effort has been put into understanding the links between life‐history traits such as body size, growth rate, size and age at maturity, and population dynamics (Ahti et al., 2020), the role of reproductive and actuarial senescence in population dynamics and population recovery remains poorly understood.

Monitoring and measuring reproductive or actuarial senescence in nature is notoriously difficult, particularly for fish, and the fishes with the most data tend to be the fishes that are the most heavily fished, therefore likely caught before senescence commences. Here, we used empirical data to parameterize an existing eco‐evolutionary model (Kuparinen et al., 2011) to overcome these obstacles (i.e., measuring senescence from a fish that has been caught and is therefore no longer in the nature contributing to the population dynamics) and to illuminate the role of senescence in fish population dynamics and population recovery under two different fishery selection schemes. As opposed to experimental or empirical studies, the simulation model allows us to control the presence and absence of senescence and explore how, all else being equal, it influences fish population dynamics in the presence and absence of fishing. We used vendace (*Coregonus albula*, Linnaeus), an economically and culturally important freshwater salmonid, as a model species. Specifically, we address the following questions: (a) How does the presence or absence of senescence influence the population dynamics of fish, in terms of asymptotic length, biomass, and number of fish in a pristine environment? And (b) how does the response to different fishery selection schemes differ depending on the presence or absence of senescence? Our results provide insights into the effects of senescence on population dynamics in pristine and harvested populations.

## 2 MATERIALS AND METHODS

To explore the role of senescence in *Coregonus albula* (Linnaeus) life‐histories and populations, we used an individual‐based model that incorporates empirical growth, fecundity, and survival data with the principles of quantitative genetics and demographic processes. The core of this mechanistic model lies in the strong negative correlation of the von Bertalanffy (vB) growth model parameters *L_∞_* (asymptotic length), and *k* (intrinsic growth coefficient, i.e., how fast the fish length is approaching *L_∞_*) (von Bertalanffy, 1938, 1949; Quince et al., 2008). Since the simulation model has been described in detail elsewhere (Kuparinen et al., 2011), we will here limit the model description to a general description of the modeling approach and the main features and additions specific to our study design. While the empirical data are from Lake Puulavesi in Central Finland, the results can be generalized to any fish with similar life‐history properties.

### 2.1 General description of the modeling approach

**FIGURE 1:**
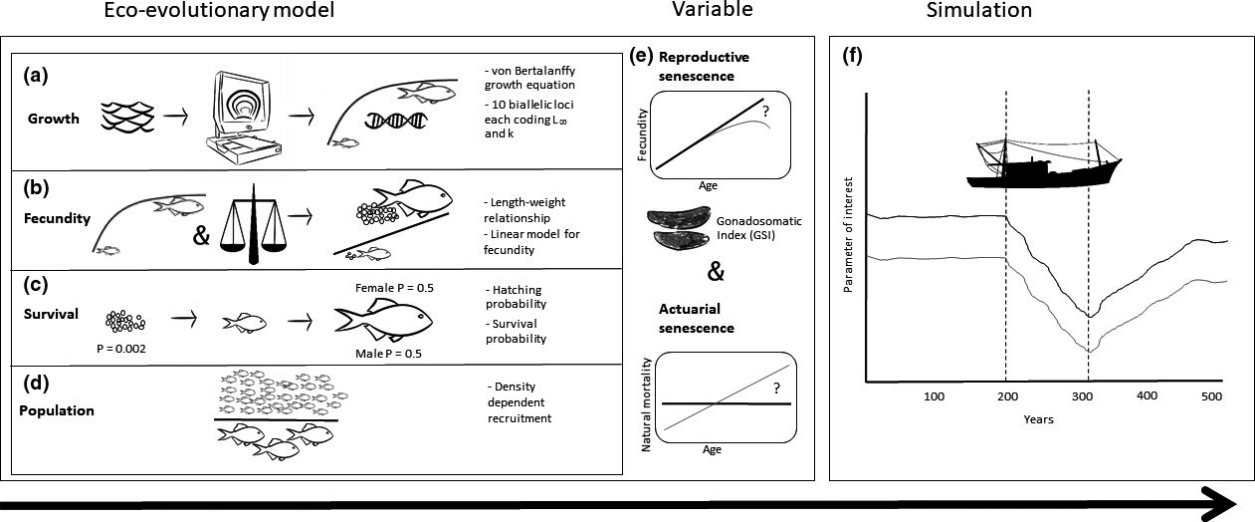
A schematic diagram of the modeling approach. (a) The fish length‐at‐age was back‐calculated from fish scales. These data were then used to fit the von Bertalanffy growth equation to model the *L*_∞_. The *L*_∞_ was set to be an evolving trait so that the genotype coding the *L*_∞_ of each individual was described by 10 diploid loci with two alleles in each, one from the mother, one from the father. (b) The length–weight relationship was specifically calculated for *C. albula* from Lake Puulavesi. Using this length–weight relationship and published empirical data on egg numbers and female weights (Karjalainen et al., 2016), we fitted a linear model for the fecundity–weight relationship, so that as the fish body size increases, so does the egg production. (c) The probability of a fertilized egg to hatch and the juvenile to survive until 2 years of age was set to *p* = .002 and the sex of the juveniles was drawn from a Bernoulli trial with a probability of 0.5. (d) The population component describes density dependency so that, for instance, at 85% of the population carrying capacity, the individual growth is reduced to 50% of that predicted by the individual's vB growth curve. Additionally, egg production was set to be density dependent so that the closer the population was to its carrying capacity, the fewer eggs were produced. (e) Reproductive senescence and actuarial senescence are the independent variables in the model. The reproductive senescence was modeled by multiplying the linear model for fecundity by the fecundity factor based on the gonadosomatic index (GSI) for the year class in question. When no reproductive senescence was modeled, only the linear model for fecundity was used. Actuarial senescence was modeled by increasing the rate of natural mortality each year, as opposed to keeping natural mortality rate constant throughout lifetime as in the scenario with no actuarial senescence. (f) Equipped with the above characters, the populations were then allowed to live for 500 years and traced at annual time steps. The first 200 years the populations lived in pristine conditions, then the populations were fished either by trawling or gillnetting for 100 years, and finally the populations were allowed to recover for 200 years

The eco‐evolutionary model includes five main components (Figure 1a–e). These are four dependent sets of variables: growth, fecundity, survival, population demographics, and an independent variable: senescence.

Life‐history traits such as size and age at maturity are thought to be controlled by many loci (Roff, 2002). In fishes, the correlation of size at maturity and *L*_∞_ is a well‐known life‐history invariant (Charnov, 1993). Thus, in the growth component (Figure 1a), we utilized empirical length‐at‐age data back‐calculated from fish scales to model the *L*_∞_. The *L*_∞_ was set to be an evolving trait so that the genotype coding *L*_∞_ of each individual was described by 10 diploid loci with two alleles in each. The alleles were inherited in the classic Mendelian way, so that each offspring received one randomly drawn allele from the mother, and one from the father. Each allele was coded as 0 or 1, and the sum of alleles across the ten loci was coupled with a normally distributed random number (mean zero) to describe phenotypic variability, and then, the sum was linearly translated to values of *L*_∞_. The standard deviation of the normally distributed random number was adjusted to yield a realistic heritability of 0.2–0.3 (Mousseau & Roff, 1987). The vB growth parameter *k* and the size at maturity were then determined based on *L*_∞_ (For more on *k* see below “Model parametrisation”). We used the empirical data to determine that the maturation size threshold was at 67% of their *L*_∞_ (mean size at 2 years of age) and no earlier than on their second autumn, which is in line with literature (Jensen, 1998; Karjalainen et al., 2016). This way, we ensured that the fish in the model will mature once they reach 67% of their *L*_∞_, but never before they reach their second autumn. Thus, fish younger than two years old, or fish smaller than 67% of their *L*_∞_ could not yet reproduce. The age at maturation was allowed to evolve, but had a restriction so that it could not go below 2 years.

The fecundity component (Figure 1b) is based on a length‐weight relationship, which was specifically calculated for *C. albula* from Lake Puulavesi. Using this length–weight relationship and published empirical data on egg numbers and female weights (Karjalainen et al., 2016), we fitted a linear model for the fecundity–weight relationship. The survival component (Figure 1c) includes an empirically based (Karjalainen et al., 2016; Marjomäki et al., 2014) probability (*p* = .002) for a fertilized egg to hatch and the juvenile to survive until 2 years of age. The sex of the juveniles was drawn from a Bernoulli trial with a probability of 0.5. Mating occurred randomly, so that for each mature female a random mate was drawn from a group of mature males. The maximum lifetime for each individual was limited to 7 years, according to local lifespan estimations in Lake Puulavesi (Marjomäki & Huolila, 1994).

The population component (Figure 1d) describes density dependency through an individual's progress along its growth trajectory. Growth continuously slows down as population density increases. For example, at 85% of the population carrying capacity (measured in biomass units, carrying capacity is 75 units), the individual growth is reduced to 50% of that predicted by the individual's vB growth curve (its *L_∞_* and *k* parameters) (see Kuparinen et al. (2011) Figure 1 for an illustration of how growth continuously slows down). This effect of population density on the individual growth was described as follows: growth time available for the individual = $\frac{e^{\left(a+b*\frac{\mathrm{BM}}{\mathrm{CC}}\right)}}{\left(1+e^{\left(a+b*\frac{\mathrm{BM}}{\mathrm{CC}}\right)}\right)}$, where *BM* = biomass in units, *CC* = carrying capacity in units, and *a* and *b* parameters describe the slope how the growth slows down. Additionally, using the same function the egg production by individual females (based on their body size) was set to be density‐dependent so that the closer the size of the spawning population was to the total population carrying capacity, the fewer eggs were produced. Inversely, the sparser the population, the more eggs were produced. Notably, while the exact numeric choices were somewhat arbitrary, implementation of these two density‐dependent mechanisms was necessary for stabilizing the population size and to keep it from exploding. The mechanisms were kept unchanged throughout the simulations and, thus, do not affect the results which are derived from the comparison of alternative simulation scenarios.

The fifth component of the eco‐evolutionary model describes senescence in its two forms: reproductive senescence and actuarial senescence (Figure 1e). The reproductive senescence was modeled by multiplying the linear model for fecundity by the gonadosomatic index (GSI, = (gonad weight/total tissue weight) × 100) for the year class in question. When no reproductive senescence was modeled, only the linear model for fecundity was used. Actuarial senescence was modeled by increasing the rate of natural mortality each year, as opposed to keeping natural mortality rate constant throughout lifetime as in the scenario with no actuarial senescence. All the other components were kept identical in the simulations (Figure 1f), but the presence and absence of reproductive and actuarial senescence were altered.

Each population in each scenario was then allowed to “live” for 500 years, and the individuals and populations were traced at annual time steps. At each annual step, the growth, reproduction, and mortality of each individual fish were simulated to get the population data for the next year. During those 500 years, the populations experienced three consecutive phases: 200 years of pristine phase, 100 years of intense fishing, and 200 years of recovery (Figure 1f). The output data of particular interest, that is, asymptotic length (*L*_∞_), biomass (*BM*), and number of fish (*N*) were collected annually. Each simulation was replicated 100 times.

### 2.2 Parametrization of the model

The empirical data were collected from Lake Puulavesi, an oligotrophic lake located in Central Finland. Its area is approximately 330 km^2^, with an average depth of 9.2 m and the deepest part reaching 62 m. The samples for vendace age and growth determination were collected from different basins of Lake Puulavesi between 1977 and 2017. While ideally the samples would have been collected from a pristine lake, no such lakes exist in Finland, and therefore, the data were collected from a lake with an average amount of fishing taking place (Marjomäki & Huolila, 2001). The model is based on empirically observed growth trajectories of vendace (total *N* = 93, female *N* = 62, male *N* = 31). The age was determined from the annuli of vendace scales located below pelvic fins. The radius of the entire scale (S) and the radius from scale focus to the annulus *i* (*S_i_*) were measured from the anterior part of the scale that was magnified (20–40×) using a microfiche reader. The ages were as follows: 3 year olds *N* = 34; 4 year olds *N* = 20; 5 year olds *N* = 37; and 6 year olds *N* = 2. Because the length at age was back‐calculated, the older the fish, the more information it provided from the previous years. Vendace is known to reach maturity usually on their second autumn, so it was here assumed that all the specimens were mature. The back‐calculation of length at age (*L_t_*) of each individual was done using Monastyrky's equation $L_{t}=L\times {\left(\frac{S_{t}}{S}\right)}^{b}$ (Monastyrsky, 1930), where *L* = the measured total length when the fish was caught, *S_t_* = the width of annulus at age *t*, *S* = radius of the entire scale, and *b* = 0.641. The value of 0.641 for the exponent *b* is an estimate from several Finnish vendace stocks (Marjomäki & Huolila, 2001).

The empirical weight data (*N* = 27) and the growth trajectories calculated above were used to calculate the length–weight relationship *W* = *a* × *L^b^* (Ricker, 1975). In this equation, *W* = fresh weight in grams (precision 0.1 g) and *L* = length in cm (precision 1 mm). The parameter *a* (scaling coefficient for the weight at length of the fish) the parameter *b* (shape parameter for the body form of the species) were calculated to be *a* = 7e−06, and *b* = 2.943. The lengths varied between 120 and 170 mm (mean 146 mm, *SD* = 8.63), and the weights varied between 12 and 27 g, (mean 18.7 g, *SD* = 3.7). The length–weight relationship is important because weight scales with fecundity and therefore plays a crucial role in population dynamics. In this particular study, it also forms the basis that reproductive senescence is modeled on.

Back‐calculated individual growth trajectories from Lake Puulavesi were summarized using a nonlinear least‐squares fit of the vB growth equation which was fit for our data *L_t_* = *L*_∞_ − (*L*_∞_ − *L*_0_)*e* ^−^ *^kt^*, where *L_t_* = length at age *t*, *L*_∞_ = asymptotic length, *L*_0_ = length at *t* = 0, and *k* = the intrinsic individual growth rate. The association between the vB parameters *L_∞_* and *k* was estimated using an empirically based linear regression model which yielded the following fit: ln(*k*) = 1.27 − 0.13 * *L*_∞_ with residual *SD* = 0.30.

In the scenarios with no reproductive senescence (i.e., how most life‐history and fisheries models describe reproduction), fecundity was based purely on the linear function for individual fecundity per gram body weight: 39.06 + 118.47 × wet mass in grams (Table 1) derived from empirical data (Karjalainen et al., 2016). For ease of comparison against the reproductive senescent scenario, we assigned a “fecundity factor” of 1 for each age group, meaning no change in fecundity with age (Table 1).

**TABLE 1:** A summary of the average lengths, median gonadosomatic indices (GSI), actuarial senescence, and reproductive senescence parameters

	Empirical data					
Age [years]	1	2	3	4	5	6+
Average length [mm]	89	114	130	137	146	147
Median GSI	25	24,747	23,184	18,841	NA	NA
	**Natural mortality (M) for each year class**					
Scenario without actuarial senescence	NA	0.257	0.257	0.257	0.257	0.257
Scenario with actuarial senescence	NA	0.2	0.258	0.314	0.372	0.428
	**Fecundity factor**					
Scenario without reproductive senescence	x 0	x 1	x 1	x 1	x 1	x 1
Scenario with reproductive senescence	x 0	x 0.990	x 0.927	x 0.754	x 0.612	x 0.498

Note

The natural mortality parameters for actuarial senescence describe how the natural mortality is spread across year classes. Natural mortality is expressed as an instantaneous rate. Fecundity was calculated as 39.060 + 118.470 * wg, where wg = weight in grams at age *t*. For populations not experiencing senescence, this was multiplied by a fecundity factor of one each year, meaning fecundity did not change with age. For populations experiencing reproductive senescence, this was multiplied by the fecundity factor shown above, simulating declining fecundity with age. For fish under the reproductive age of 2, the fecundity factor was zero, and therefore, no reproduction took place. The fecundity factor describes the relative change in gonadosomatic index (GSI) over age classes.

Karjalainen et al. (2016) showed an age‐dependent decrease in the relation of body and gonad weight which we used to calculate gonadosomatic index (GSI), a pattern that could be indicative of reproductive senescence given it has been used as a proxy in other studies (Benoît et al., 2018; Hendry et al., 2003). We used this GSI as a proxy for reproductive senescence (Table 1). As we only had GSI data for fish up to four years old, the GSI for 5‐, 6 and 7‐year‐old fish was linearly extrapolated from the existing data. We used linear extrapolation, because we are interested in the mechanistic changes in a population, and not specifically only in vendace. Instead of using the absolute GSI values to describe reproductive senescence in the model, we standardized the effect of reproductive senescence so that the GSI for age group 1 was set to be the baseline and have a fecundity factor of 1 (i.e., no change, same as the nonsenescent population), and the following age groups from 2 to 6 were assigned a fecundity factor proportional to that of age group 1. The fecundity factor was calculated by dividing the GSI of age group 1 by the GSI of the age group in question, so for instance to get the fecundity factor for age group 4 would be as follows: GSI for age group 1/GSI for age group 4. The linear function for fecundity (described above) was then multiplied by the appropriate fecundity factor for each age group (Table 1) to model reproductive senescence. This way, as the fish ages, its reproductive output declines.

When actuarial senescence was not modeled, the instantaneous natural mortality rate was coded to be an age‐independent constant of *M* = 0.257 in all adult age groups (Table 1).

To model actuarial senescence, we coded an instantaneous natural mortality rate (*M*) that increases with age. The senescence scenario was modeled so that a baseline natural mortality of *M* = 0.2 was set for 2 year olds, and the added mortality rate per each age group was adjusted to proportion from Marjomäki (2005) and is shown in Table 1. The difference between natural mortality imposed by actuarial senescence and fishing mortality is that natural mortality as a result of actuarial senescence increases with age, while the fishing mortality is size‐dependent.

To allow for a careful investigation of the resulting demographic structures, the natural mortality at the population level was set to be identical in all scenarios. This means that the total natural mortality over time in all scenarios is the same, but for populations with no actuarial senescence the mortality rate was unchanged over age classes, and for the populations with actuarial senescence present, the mortality increased with age. So, whether the natural mortality rate remained unchanged over age classes or increased with age, the total realized natural mortality for a population over time was equal, only the distribution among age classes differed.

An increase in natural mortality following sexual maturity is an important trade‐off in life‐history evolution (Kuparinen et al., 2011). To take this into account, and to add biological realism in the model, a survival cost of maturation, that is, increased mortality rate after having become sexually mature was added to the natural mortality rate in every scenario, for every maturing fish. The survival cost of maturation was estimated to be the increase in mortality rate from age group 1 to age group 2 as per Marjomäki (2005), and this was applied once in every scenario, whether actuarial or reproductive senescence was modeled or not.

### 2.3 Simulation design

The initial starting population was 2,000 individuals and the initial body size was 4 cm. The population size and the initial body size were selected due to model optimization and play no role in the results of the study. A burn‐in simulation of 1,000 years was then run for each scenario. All populations reached a state of ecological stability in approximately 600 years. The population carrying capacity is 75 units. The populations leveled off at around 56–61 units. One hundred ecologically and evolutionarily stable populations were saved for all scenarios, and these populations were then sampled to be used as the starting population in further simulations.

Simulations were run for 500 years. The population was kept in a pristine equilibrium state for 200 years before fishing was simulated for a period of 100 years. Fishing started in year 200 and ceased in year 300, and populations were then allowed to recover for 200 years. Vendace is traditionally fished by seining and trawling (Salmi, 1998), which means that the retention probability increases with the size of the fish to a certain size and is constant after that. To mimic seining or trawling selection and to describe length‐dependent gear selectivity in the population, we used a logistic curve $r\left(l\right)=\left(\frac{\exp(a+bl)}{1+\exp(a+bl)}\right)$, where *r*(*l*) = the retention probability of a fish of length *l*, and *a* = −9 and *b* = 0.85 are shape parameters, so that 50% retention probability is reached at length −a/b (Kuparinen et al., 2009). We also ran separate simulations for gill net fishing by describing a dome‐shaped selectivity curve (for reasons why we assume gillnetting to have a dome‐shaped selectivity curve, see (Kuparinen et al., 2009)) $r\left(l\right)∼\exp\left(‐\frac{{\left(l‐\mu \right)}^{2}}{2\sigma }\right)$, where *r*(*l*) = the retention probability of a fish of length *l*, *µ* = 12 (fish length in cm at which the selection curve peaks), and *σ* = 0.5 (standard deviation describing the width of the curve around its peak). For simplicity hereafter, when we discuss seining or trawling, a logistic selection curve is assumed, and when we discuss gillnetting, a dome‐shaped selection curve is assumed. Regardless of the fishing method, the fishing mortality (*F*) of the fully selected size class was set to 0.7, which is considered a realistic level of magnitude for intensively fished populations (Viljanen, 1986). The fishing mortality in terms of biomass was kept identical for the senescent and nonsenescent scenarios. All scenarios were explored across pristine, harvest and recovery periods over 500 years. We created 100 independent replicates for each scenario.

All simulations and analyses were conducted using R version R‐4.0.3 (R Core Team, 2018). The code is available in the Supplementary material.

## 3 RESULTS

**FIGURE 2:**
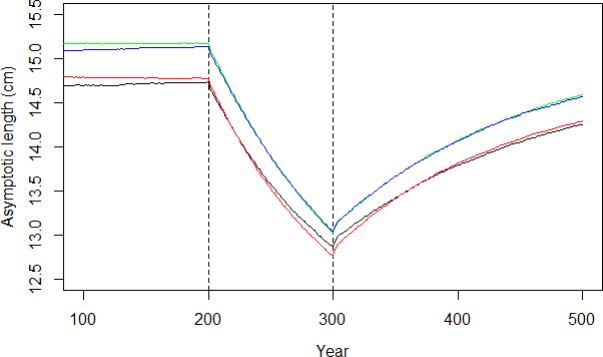
The mean of the asymptotic length (cm) of fish over 500 years (first hundred years not shown). The dashed lines denote the start (year 200) and end (year 300) of fishing. The solid lines denote the asymptotic length mean in hundred replicated scenarios. The black line describes a scenario with reproductive and actuarial senescence, red line a scenario with actuarial senescence only, blue line a scenario with no senescence, and green line a scenario with reproductive senescence only

Populations in all scenarios had reached an equilibrium and therefore showed only minor temporal fluctuations in any of the population parameters before fishing commenced in year 200 (Figure 2). However, scenarios including actuarial senescence consistently differed from those that did not include actuarial senescence. These differences were seen before, during, and after fishing in all output variables investigated. Given that actuarial senescence appeared to be the major cause of the differences (Figure 2), likely due to the relatively low reduction in reproductive output with age (Figures S1 and S2), we focus most of the present work on two instead of four scenarios: a scenario with both reproductive and actuarial senescence and a scenario with no senescence. This is because the GSI is based on empirical data (Karjalainen et al., 2016), so comparing a scenario based on actual empirical data to a scenario where both reproductive and actuarial senescence are assumed completely absent enables us to investigate the potential differences between models that include and do not include empirical life‐history characteristics of senescence. Additionally, reproductive and actuarial senescence are known to be linked (Kirkwood & Shanley, 2010), so exploring either both types of senescence together or none at all is biologically more relevant than separating the senescence types.

No trends toward different life‐history strategies were seen between the different simulation replicates in either the scenarios with senescence (Figure S3) or without senescence (Figure S4).

### 3.1 Asymptotic length, body size, and age

**FIGURE 3:**
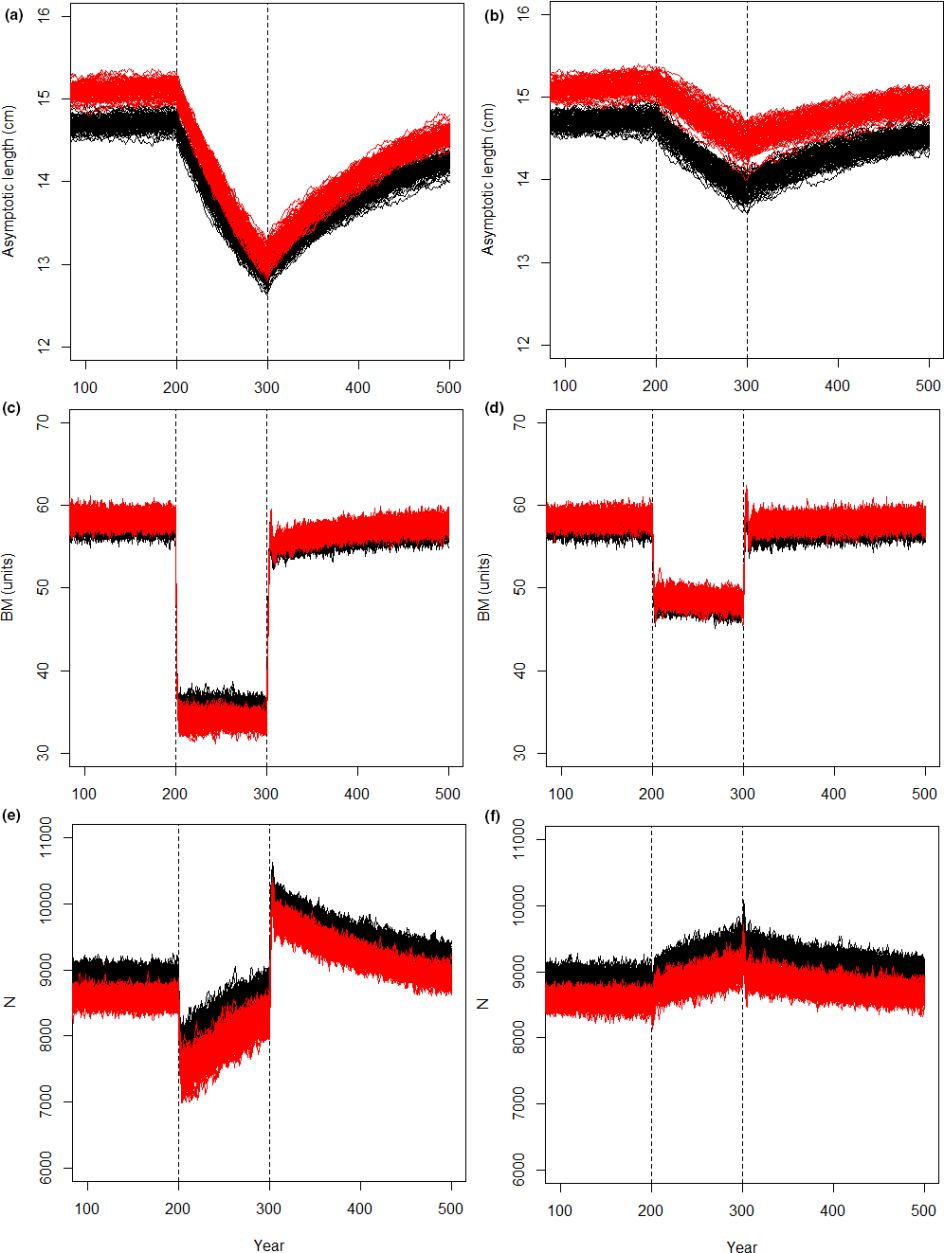
Results for the (a, b) asymptotic length (cm), (c, d) biomass (units), and (e, f) the number (*N*) of fish. In (a, c, and e), the fishing simulated trawling (logistic selection) and in (b, d, and f) the fishing simulated gillnetting (dome‐shaped selection). The solid black lines represent hundred independent replicates of the scenario with senescence, the red lines represent hundred independent replicates of the scenario with no senescence present. The dashed lines denote the start (year 200) and end (year 300) of fishing

**FIGURE 4:**
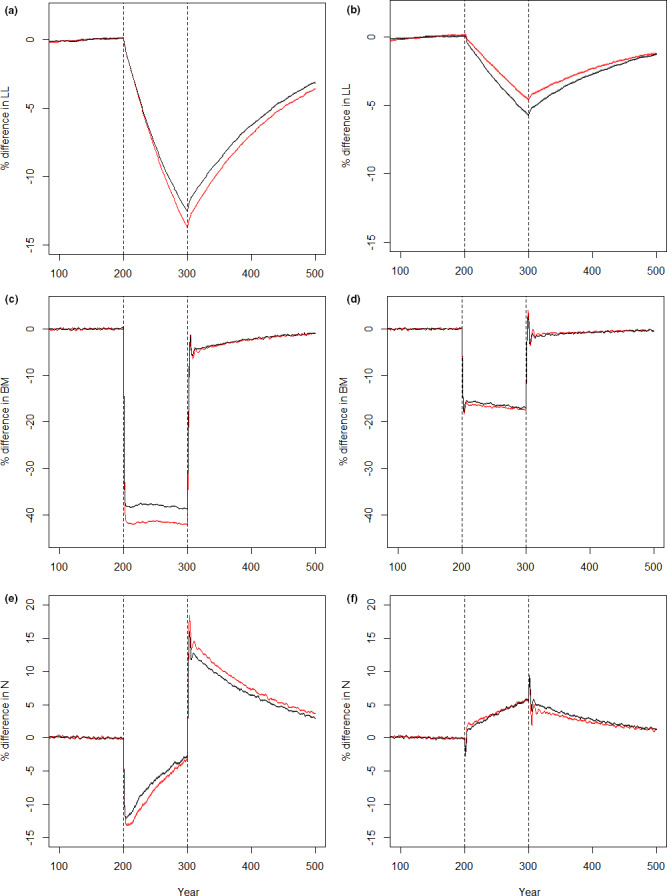
The relative percentage change (a, b) asymptotic length (cm), (c, d) biomass (units), and (e, f) the number (*N*) of fish. In (a, c, and e), the fishing simulated trawling (logistic selection) and in (b, d and f) the fishing simulated gillnetting (dome‐shaped selection). The change has been scaled so that years 1–100 were considered as the starting point and given a value of 0. Changes in all of the parameters (asymptotic length, *BM*, *N*) after that are relative changes compared to years 1–100. The black lines denote a scenario with senescence, and the red lines denote a scenario without senescence. The dashed lines denote the start (year 200) and end (year 300) of fishing. Given the scale of the *Y* axis, the 95% confidence intervals are virtually invisible

**FIGURE 5:**
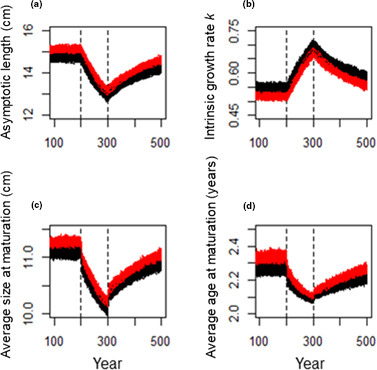
Results for the (a) asymptotic length (cm), (b) intrinsic growth rate *k*, (c) average size (cm) at maturation, and (d) average age (years) at maturation over hundred years (first hundred years not shown). The black lines represent hundred independent replicates of the scenario with senescence, and the red lines represent hundred independent replicates of the scenario with no senescence present. The dashed lines denote the start (year 200) and end (year 300) of fishing

The populations with senescence had a consistently lower *L*_∞_ than those with no senescence. For both the senescent and nonsenescent scenarios, fishing caused a decline in *L*_∞_ (Figure 3a,b), and the decline caused by trawling (Figure 3a) was larger than the decline caused by gillnetting (Figure 3b), regardless of the presence of senescence. However, the type of fishing played a role in the relative change within a scenario. The senescent scenario had a smaller decline in *L*_∞_ than the nonsenescent scenario when trawled (Figure 4a). The opposite occurred when gillnetting was applied: The senescent scenario had a larger drop in *L*_∞_ as a result of dome‐shaped fishing compared to the nonsenescent scenario (Figure 4b). When fishing was ceased after 100 years, *L*_∞_ started to increase slowly in all scenarios, but in none of the scenarios did the *L*_∞_ recover back to the level prior to fishing. Associated changes in the vB growth parameter *k*, and average size and age at maturation are shown Figure 5a,b,c,d, respectively.

**FIGURE 6:**
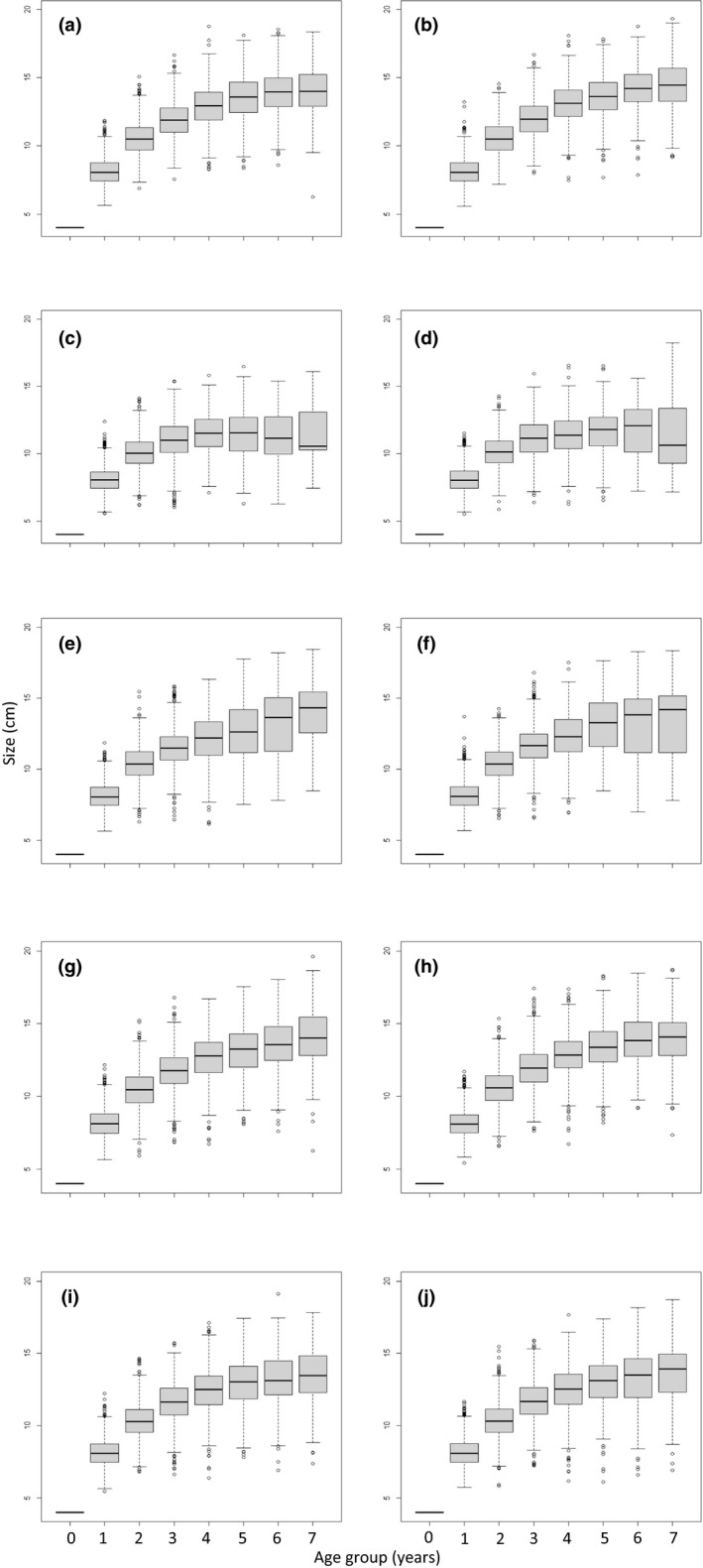
The fish size (cm) distribution by age groups. The different panels are as follows: (a) Senescence scenario before fishing, (b) No senescence scenario before fishing, (c) Senescence scenario during fishing (logistic selection), (d) No senescence scenario during fishing (logistic selection), (e) Senescence scenario during fishing (dome‐shaped selection), (f) No senescence scenario during fishing (dome‐shaped selection), (g) Senescence scenario after fishing (logistic selection), (h) No senescence scenario after fishing (logistic selection), (i) Senescence scenario after fishing (dome‐shaped selection), and (j) No senescence scenario after fishing (dome‐shaped selection)

Investigation into the variation among individuals in the evolved asymptotic length showed no alternative life‐history strategies at any stage before, during, or after fishing, and in both the senescent and nonsenescent scenarios the asymptotic length distribution was approximately normally distributed (Figure S5). The variation in fish body size (not asymptotic length) (Figure S6) and age structure (Figure S7) revealed a shift toward smaller and younger individuals during fishing, much like seen in Figures 3 and 4 for the average asymptotic length. When looking at the size and age distribution together (Figure 6), it can be seen that in the senescent scenarios both trawling (Figure 6c,d) and gillnetting (Figure 6e,f) led to a slight reduction in the variation of body size in the older age groups, compared to the scenario with no senescence (Figure 6d for trawling, Figure 6f for gillnetting). One hundred years after fishing was ceased, these differences had largely diminished (Figure 6g‐j).

### 3.2 Biomass

In the absence of fishing, whether pristine or recovery phase, the scenario with senescence produced a lower biomass than the scenario without senescence (Figure 3c,d). When fishing pressure was applied the population biomass declined in both scenarios. Trawling (Figure 3c) caused a larger drop than gillnetting (Figure 3d). However, when trawled, the population with senescence maintained a higher biomass than the one without senescence (Figure 3c). In the gillnetting scenario, the senescent population had a slightly lower biomass during fishing compared to the nonsenescent population (Figure 3d).

Regardless of the type of fishing, the relative drop in biomass for populations with senescence was smaller than for those with no senescence (Figure 4c,d). When trawling was applied, the level of biomass stayed relatively constant for both senescent and nonsenescent scenarios (Figure 4c). However, gillnetting caused a sharp decline in biomass then a sharp increase and then a slow, continuous decline for both scenarios (Figure 4d). The decline did not level off at any point during hundred years of fishing. When fishing was ceased, all scenarios experienced a rapid increase in biomass with an initial peak as large fish were again free from harvest and then a sharp drop as density‐dependent processes started controlling the population again. These peaks and drops were larger in the gillnetting scenario than in the trawling scenario. While all scenarios eventually settled to little variation and a slow, increasing trend, in two hundred years of recovery, no population had recovered to the prefishing levels.

### 3.3 Number of fish

The number of individuals (*N*) was consistently higher for populations with senescence, than for those without, regardless of fishing type or the presence or absence of fishing (Figure 3e,f). The start of trawling caused a rapid initial decline in the number of fish, but as trawling continued, the *N* increased in both senescent and nonsenescent scenarios, and however, it never reached the prefishing level (Figure 3e). This was different from gillnetting, which caused a steady increase in the *N* during fishing, above the prefishing levels (Figure 3f). As fishing was ceased, the populations in trawling scenarios experienced a rapid initial increase in *N*, and then a declining trend. When gillnetting was ceased, it caused a slow decline in the number of fish. No scenario reached the prefishing level in two hundred years of recovery.

In both trawling and gillnetting scenarios, the relative change in the number of fish was smaller for the senescent scenario, compared to the nonsenescent scenario (Figure 4e,f). However, as the fishing continued, the difference between the senescent and nonsenescent scenarios declined.

## 4 DISCUSSION

Changes in fish life‐history characters are known to scale up to the ecosystem level (Kuparinen et al., 2016). At the same time, many collapsed fish populations have not recovered even after significant reductions in fishing pressure or are recovering slower than expected (Hutchings & Reynolds, 2004). The causes for the lack of recovery are complex (Swain et al., 2011), but a careful look at the life‐history characters of different species may enable us to better predict the response of fish populations to fishing. Our individual‐based eco‐evolutionary simulation model sheds light on how often ignored life‐history characters, namely, reproductive and actuarial senescence, may affect fish population dynamics under different fishing selection regimes. While the asymptotic length of senescent and nonsenescent fish responds to different fishing selection regimes with a different magnitude, the population level consequences of senescence might be partially density dependent (Graves & Mueller, 1993). During high external mortality, it might be better to invest in reproduction rather than grow large. Indeed, high rate of external mortality is expected to accelerate the rate of senescence (Williams, 1957) and as a trade‐off potentially select for earlier reproduction.

An interesting connection raises from the potential link between senescence, other life‐history traits associated with senescence, and fisheries induced evolution. In scenarios with senescence, populations evolved to have a smaller asymptotic length and coupled with this was a higher intrinsic growth rate, as well as evolution toward younger age at maturation. These trends were present both when there was no fishing, and therefore, density‐dependent processes regulated the population size, and when fishing had relaxed the population from strong density‐dependent competition. Both extrinsic mortality (fishing) and intrinsic mortality (actuarial senescence) led to a declining asymptotic length. This may indicate the presence of a trade‐off between increased investment in growth and/ or reproduction early in life (as lower asymptotic length was associated with higher growth rate and maturation at a younger age) and decreased survival later in life. The well‐known effects of fishing‐induced evolution, that is, selection toward smaller size, smaller size at maturation, and higher growth rate (Heino et al., 2015; Hunter et al., 2015; Uusi‐Heikkilä et al., 2015) may therefore enhance the trade‐offs associated with senescence and the evolution of life‐history traits. Additionally, if increased allocation of resources to reproduction early in life is associated with decreased survival later in life (Kirkwood & Rose, 1991), then fishing‐induced evolution could be indirectly promoting the evolution of senescence through selecting for smaller size and age at maturity.

The different response of senescent and nonsenescent scenarios to trawling and gillnetting shows that the presence or absence of senescence can affect the magnitude of the change in life‐history traits in response to fishing. The evolutionary response to trawling in terms of asymptotic length was smaller in the senescent scenario than in the nonsenescent scenario. It could be speculated that the smaller asymptotic length of the senescent scenarios “pre‐adapts” the population to the consequences of fishing by trawling. As the fish have a smaller asymptotic length to start with, and trawling removes specifically the large fish, the relative change in their asymptotic length is therefore not as large as in the scenarios with no senescence where the asymptotic length is higher to start with. Additionally, as the fish in the senescent scenario matured younger and smaller already before any fishing‐induced evolution took place, the evolutionary pressure for change is not as high as it would be for the nonsenescent scenario where fish are larger and mature larger and older.

Gillnetting, on the other hand, allows some of the larger individuals to escape, and as a result, the evolutionary response in asymptotic length is smaller than in the trawling scenario. However, the magnitude of the response is the opposite from the trawling scenario: When gillnetting was applied, the senescent population had a stronger response in asymptotic length than did the nonsenescent population. On the one hand, given that the nonsenescent scenario would have more old and large fish alive than the senescent scenario, those old and large individuals could help keep the asymptotic length in the population higher than in the senescent scenario. In the senescent scenario, on the other hand, the proportion of smaller fish was higher to start with, making the asymptotic length decline more in response to fishing as more smaller fish would pass on more genes for lower asymptotic length. These different responses of senescent and nonsenescent scenarios to different fishing regimes highlight the importance of considering life‐history traits when managing fish populations that are exposed to different types of fishing selection, especially as these differences are seen even when the variation in age and size structures between scenarios are very small.

The changes in asymptotic length, and associated changes in growth rate, and size and age at maturity translated to changes in population level variables. As the carrying capacity of both senescent and nonsenescent populations was the same, and the asymptotic length of the fish decreased as a result of senescence and/ or fishing, the senescent population could contain a higher number of fish through pristine, fishing, and recovery phases. However, the response of the senescent and nonsenescent populations in terms of biomass differed during fishing, and the type of fishing affected the response.

In absolute terms, the senescent population maintained a higher biomass during trawling than the nonsenescent population. Our simulation allowed for control over the fishing mortality, and the catch in terms of biomass was set identical for the senescent and nonsenescent populations. Before the start of fishing, the population with senescence had a lower biomass than that of the nonsenescent population. Since the absolute biomass of the catch is the same in both populations, this means that the proportional catch from the senescent population (with initially lower biomass) is higher than the catch from the nonsenescent population (which had a higher biomass initially). Therefore, the lower asymptotic length of the senescent population did not lead to them being less likely to get caught, but indeed the opposite. Regardless of being more likely to get caught, the senescent population maintained a higher number and higher biomass than the nonsenescent population in trawling.

The explanation for the higher biomass and the higher number of fish in the senescent scenario during trawling compared to the nonsenescent scenario is likely in the life‐history trade‐offs. The population with senescence has evolved to have a lower asymptotic length, and therefore, they mature and start reproducing younger and at a smaller size. Fishing as a source of external mortality pushes the age and size at maturity even younger and smaller, so the higher biomass and number of fish is likely maintained by this earlier reproduction and not lesser fishing mortality. This is also reflected in the fact that compared to the nonsenescent scenario, the senescent scenario had a smaller size variation of old individuals, but the size variation of young individuals did not differ from that in the nonsenescent scenario. Consequently, due to the carrying capacity restriction, the truncated size structure in the older age groups for the senescent scenario allowed for more variation in the size structure of the younger age groups, and therefore potentially higher reproductive output, given that body size influences fecundity.

Gillnetting presents a different kind of selection curve than trawling. While the logistic selection curve of trawling allows virtually no escape of the larger fishes, the dome‐shaped selection curve of gillnetting selects the mid‐sized and allows the escape of small and large fish. This kind of selection curve leads to a less skewed population in terms of size and age (Figure 6). While the fish in the senescent scenario were smaller, there were more of them than in the nonsenescent scenario. As a result, the responses of the senescent and the nonsenescent scenarios to gillnetting in terms of biomass did not differ as much as they did to trawling. Like in the trawling scenario, the increased reproduction of younger and smaller fish is likely to drive the relatively higher biomass of the senescent population.

Based on the evidence for the presence of senescence in fish (Benoît et al., 2018; Beverton et al., 2004; Carlson et al., 2007; Comfort, 1960, 1963; Gerking, 1957; Hendry et al., 2004; Morbey et al., 2005; Patnaik et al., 1994; Reznick et al., 2004, 2006; Terzibasi Tozzini et al., 2013; Uriarte et al., 2016; Woodhead, 1998; Woodhead & Ellett, 1966, 1967, 1969a, 1969b; Žák & Reichard, 2020), taking senescence into consideration in fisheries stock assessments could improve the accuracy of stock assessment and success in management. As described by Le Bris et al. (2015), fisheries models that predict population dynamics often assume that individual fecundity increases with the increasing size of fish. These models are particularly sensitive to variations in the fecundity–mass relationship (Le Bris et al., 2015). Therefore, for species that undergo senescence, estimates of fecundity that ignore senescence may prove to be incorrect.

Similarly, due to lack of age‐specific natural mortality data, typical fisheries models assume a constant rate of natural mortality regardless of the age and size of the fish, or a rate of natural mortality that scales with body size raised to a negative power (summarized in Gislason et al., 2010), thereby assuming a decreased rate of natural mortality as the individual grows and ages. While accurate for many species, for species experiencing senescence, ignoring increasing natural mortality with age could lead to unrealistically low mortality estimates, as well as skewed fecundity estimates given that the old and large individuals may not be there to reproduce. Recruitment and natural mortality are the basic building blocks of stock assessment, and therefore ignoring the ways that senescence can change them could lead to biased estimates of fish population sizes. Inaccuracies in stock assessment models, whether related to reproductive capacity or mortality rates, may risk the sustainability of fishing.

Senescence can mask changes in life‐history responses to fishing. As demonstrated in the present study, the presence or absence of senescence can affect how the population responds to different fishing selections regimes: while trawling reduced the asymptotic length of nonsenescent population more, gillnetting reduced the asymptotic length of the senescent population more. Depending on the fishing method in question, the magnitude of change in life‐history characters may be higher or lower than anticipated depending on whether senescence is present or not. As a result, the population level response will change too. Failure to consider senescence as a fish life‐history trait with trade‐offs and population level consequences will hinder our progress in understanding fish population resiliency.

The shortage of pristine environments brings limitations to all ecological studies. This was no different in the current work. Our model was parametrized with data from a fished population because it is impossible to collect data from a pristine population given that as soon as the data are collected the population is no longer pristine. It is likely, however, that this has no effect in the results of the study, as the study focuses on large mechanisms instead of absolute numbers, and most populations of concern for fisheries management are also fished. Similarly, mathematical simulation modeling allows for data generation based on collected empirical data, and the relatively low sample size of 93 individuals was therefore sufficient for the present work. By their very nature models are simplifications of the real world, and before used to predict phenomena in other populations, the life‐history characters of such populations should be cautiously considered.

## CONFLICT OF INTEREST

The authors declare no conflict of interests.

## AUTHOR CONTRIBUTIONS

**Pauliina A. Ahti:** Conceptualization (equal); formal analysis (lead); writing‐original draft (lead); writing‐review & editing (lead). **Silva Uusi‐Heikkilä:** Supervision (equal); writing‐review & editing (supporting). **Timo J. Marjomäki:** Resources (equal); writing‐review & editing (supporting). **Anna Kuparinen:** Conceptualization (equal); funding acquisition (equal); supervision (equal); writing‐review & editing (supporting).

## Supporting information

Fig S1

Fig S2

Fig S3

Fig S4

Fig S5

Fig S6

Fig S7

Appendix S1‐7‐Legend

Supplementary material

## Data Availability

The code used for the simulations is accessible in Dryad at https://doi.org/10.5061/dryad.c866t1g7j.
