# Supplementary figures and images for: Age is not just a number—Mathematical model suggests senescence affects how fish populations respond to different fishing regimes
Source: Ecol Evol. 2021 Sep 7;11(19):13363–78. doi: 10.1002/ece3.8058 (PMC8495815; doi:10.1002/ece3.8058)

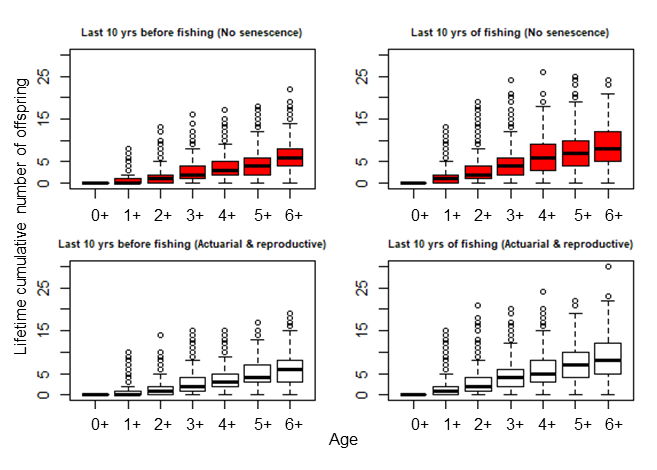

Supplement: Supplementary file 1 — Fig S1 [file ECE3-11-13363-s006.png]

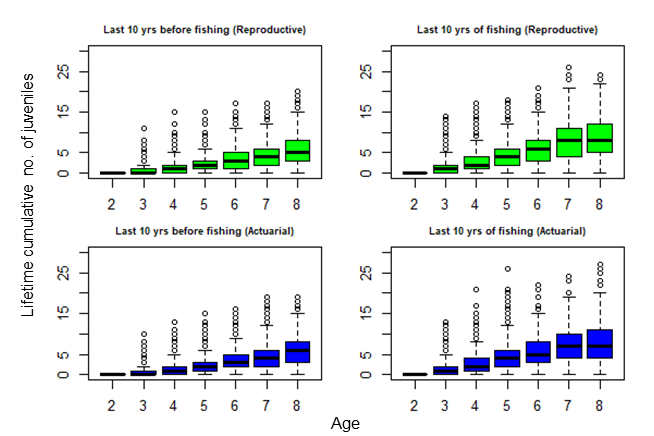

Supplement: Supplementary file 2 — Fig S2 [file ECE3-11-13363-s007.png]

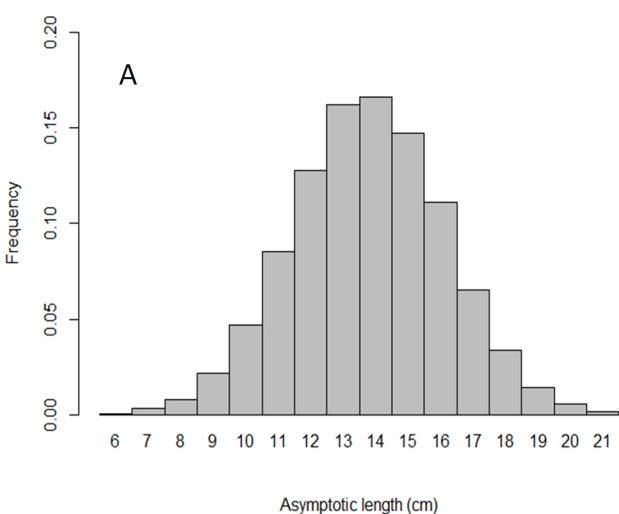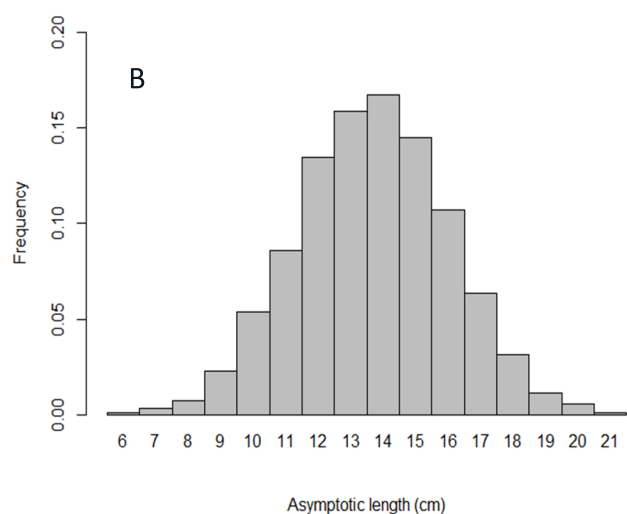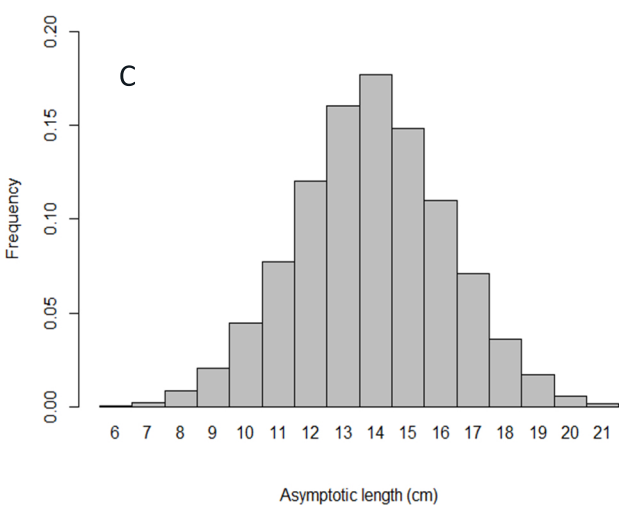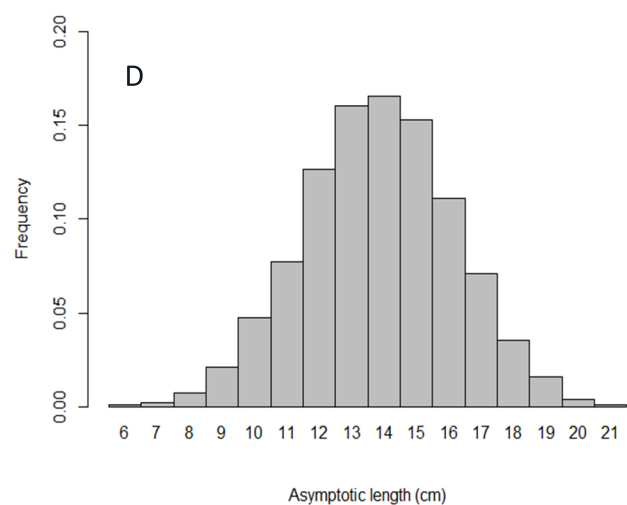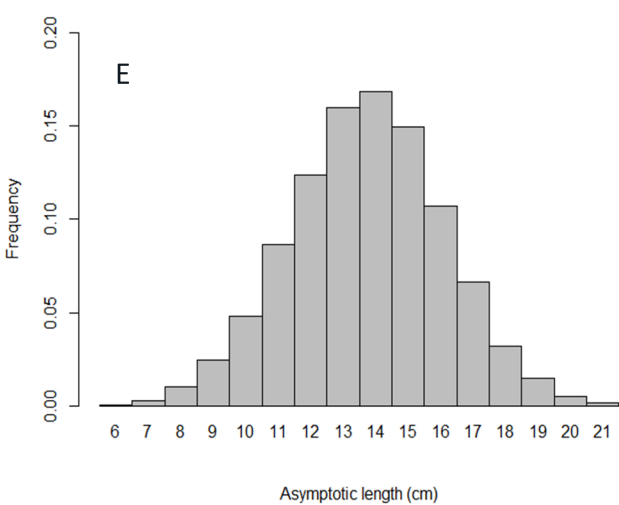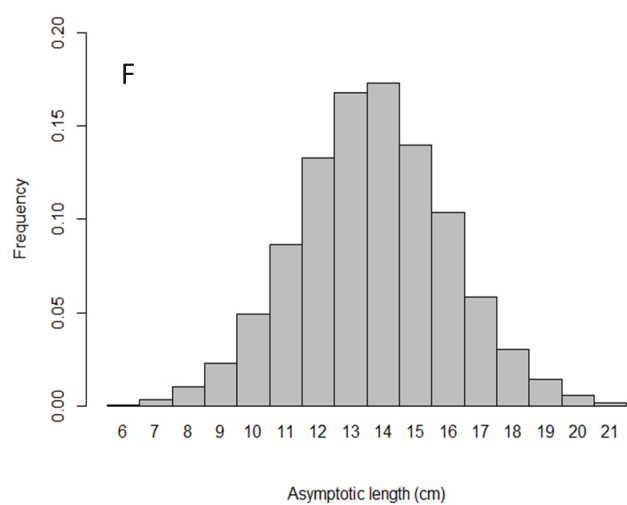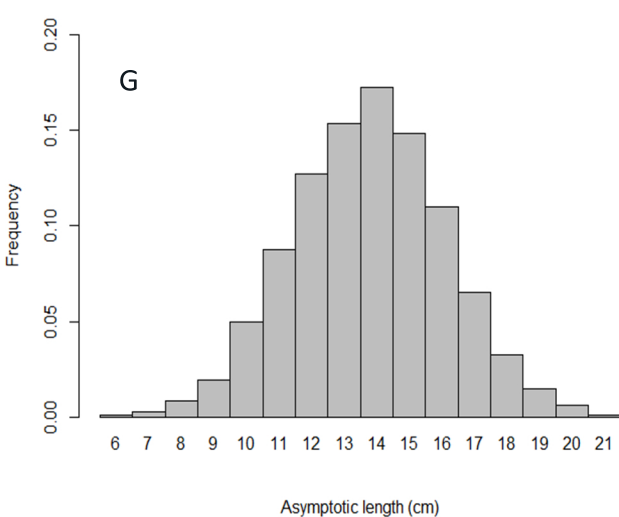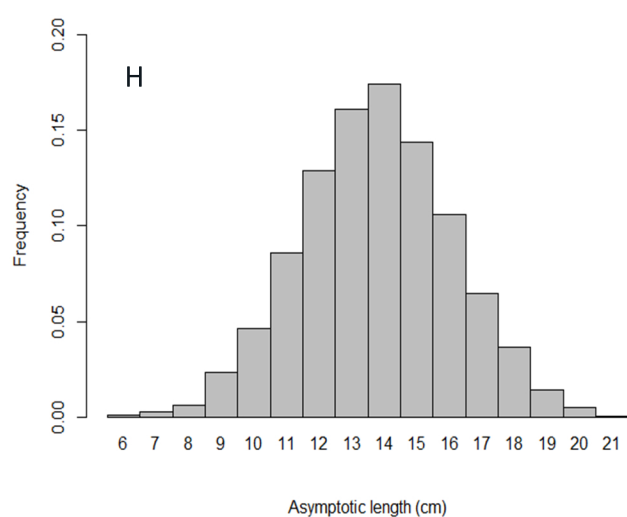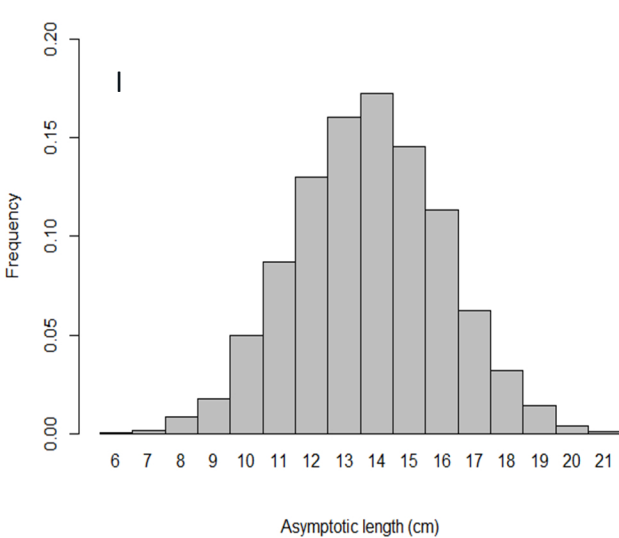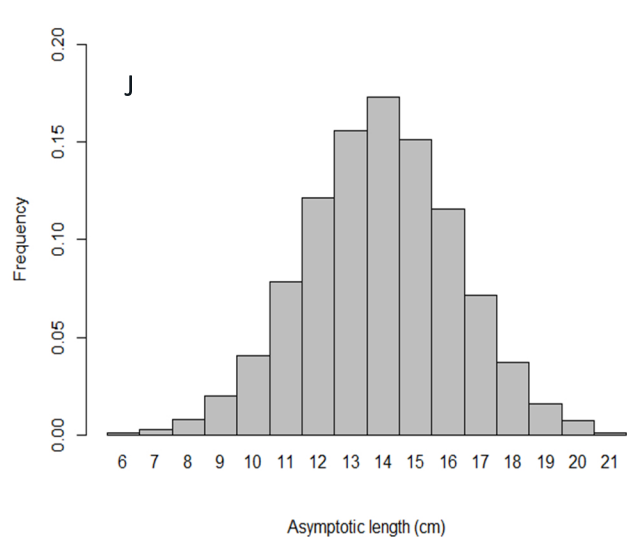

Supplement: Supplementary file 3 — Fig S3 [file ECE3-11-13363-s002.pdf]

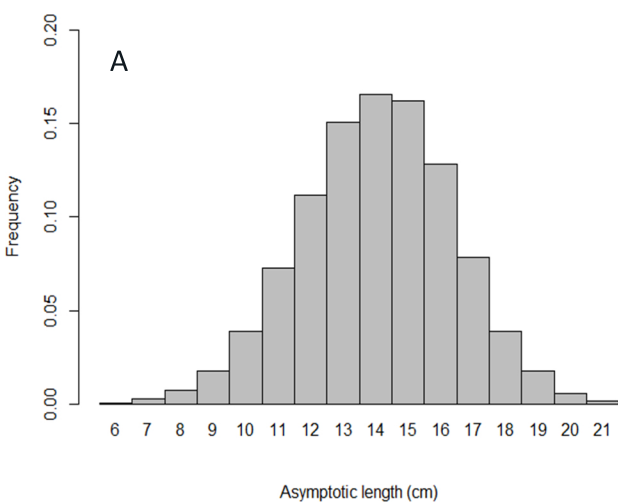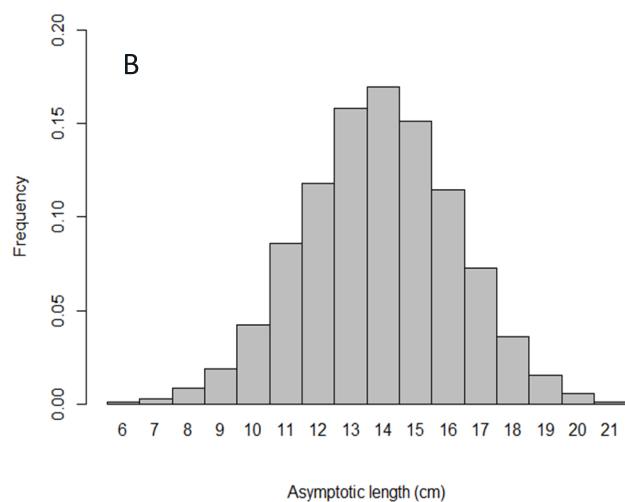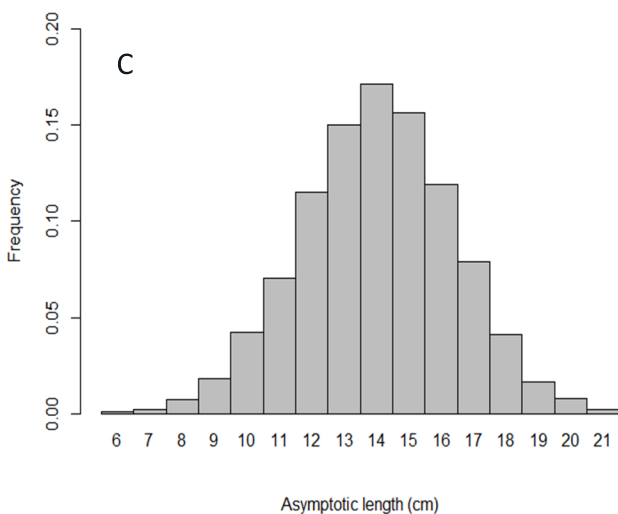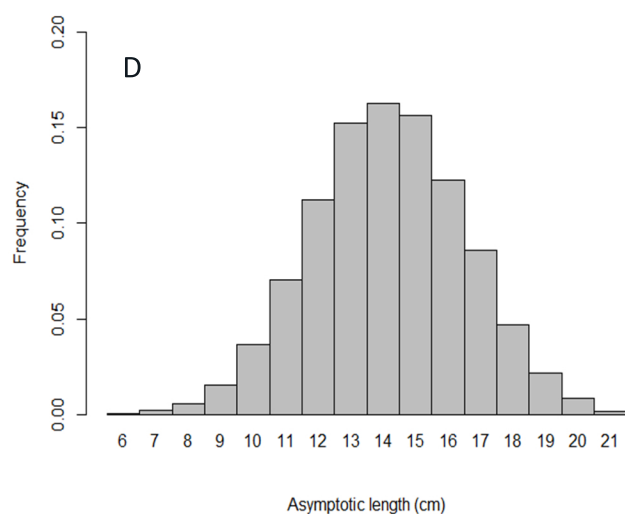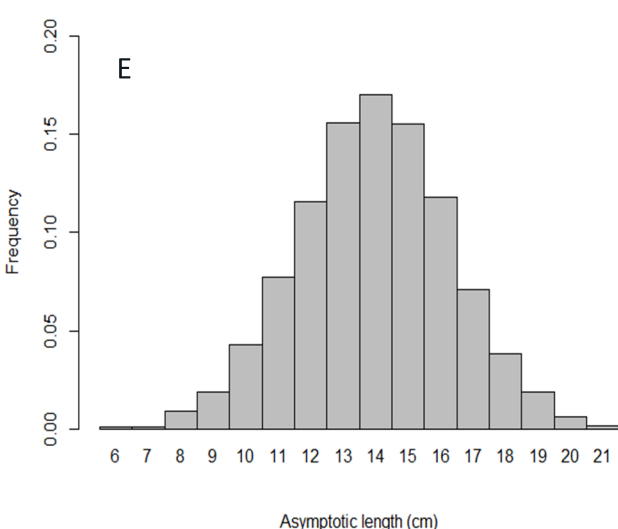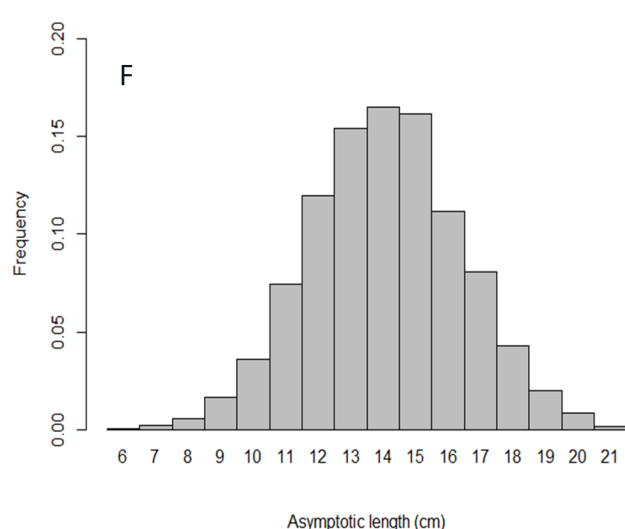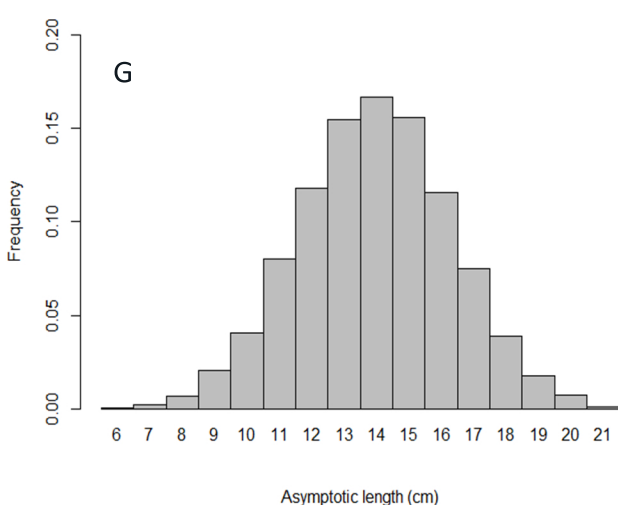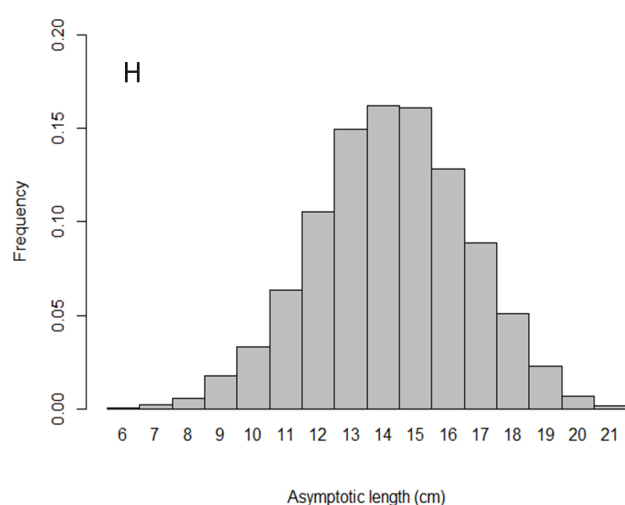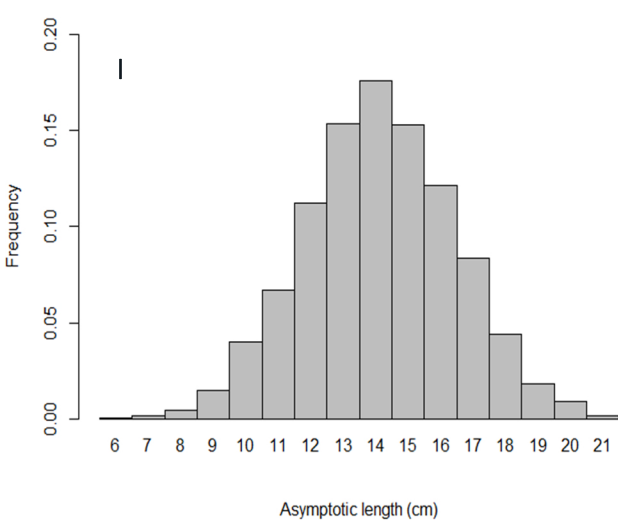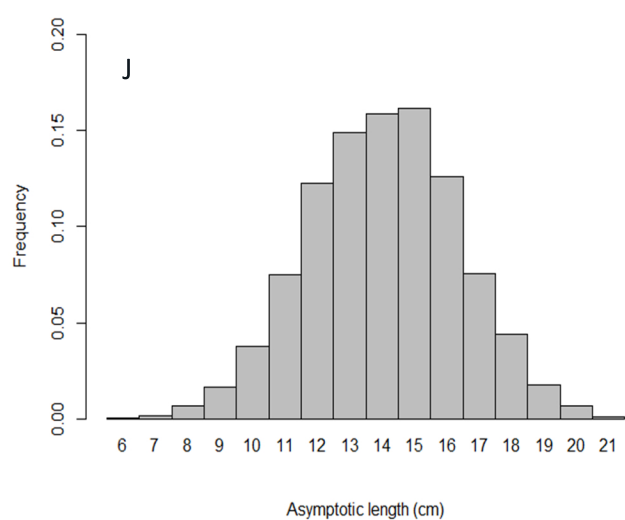

Supplement: Supplementary file 4 — Fig S4 [file ECE3-11-13363-s009.pdf]

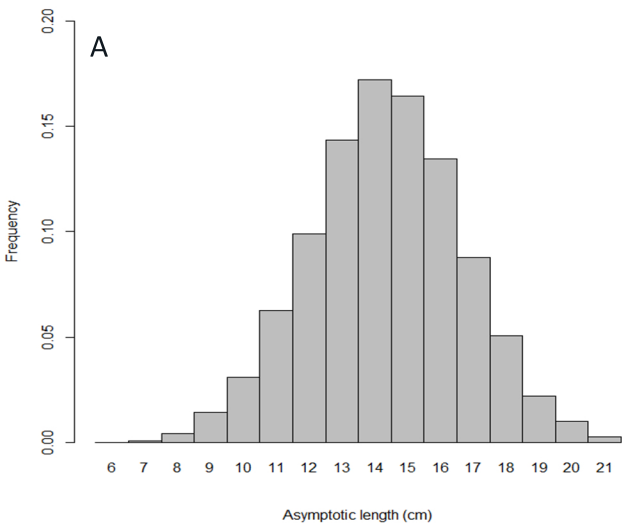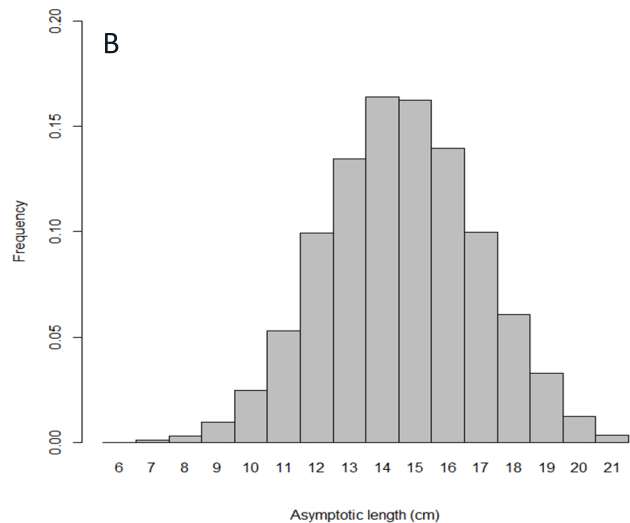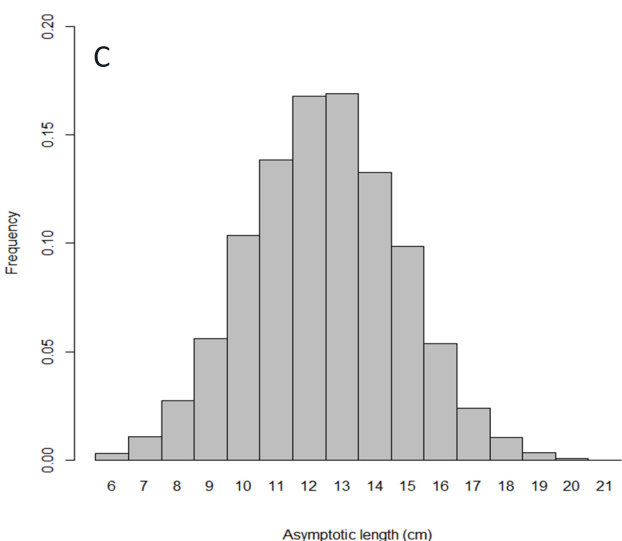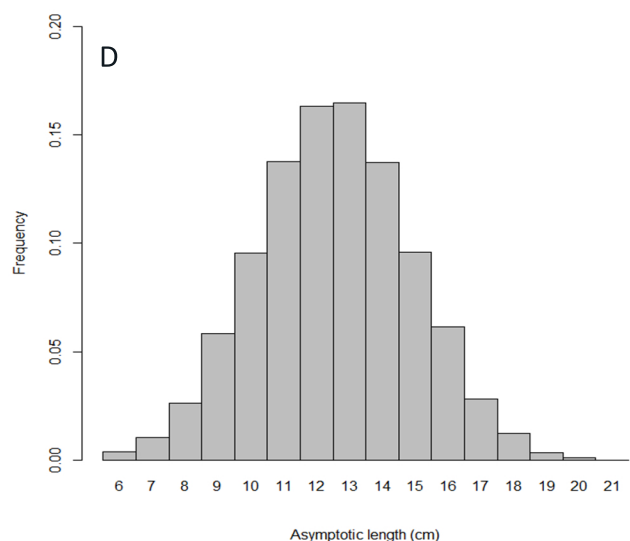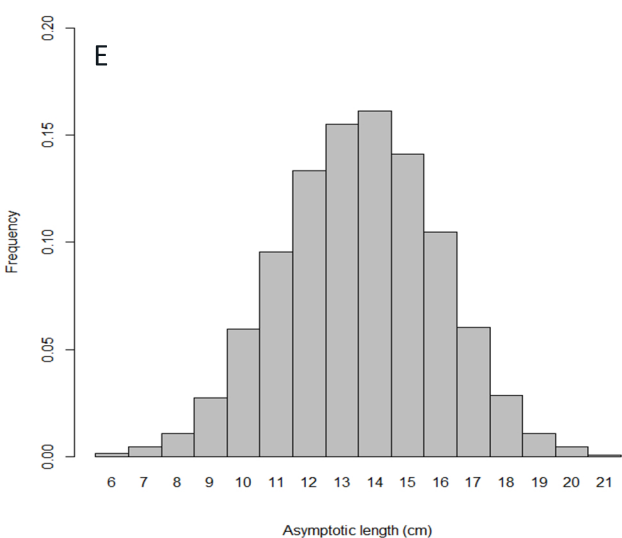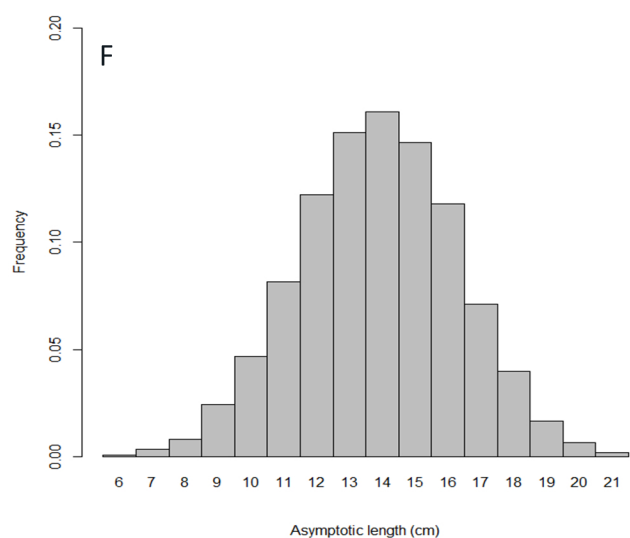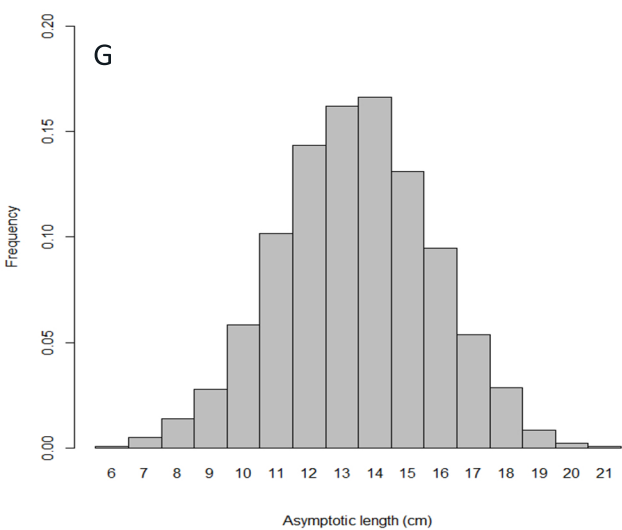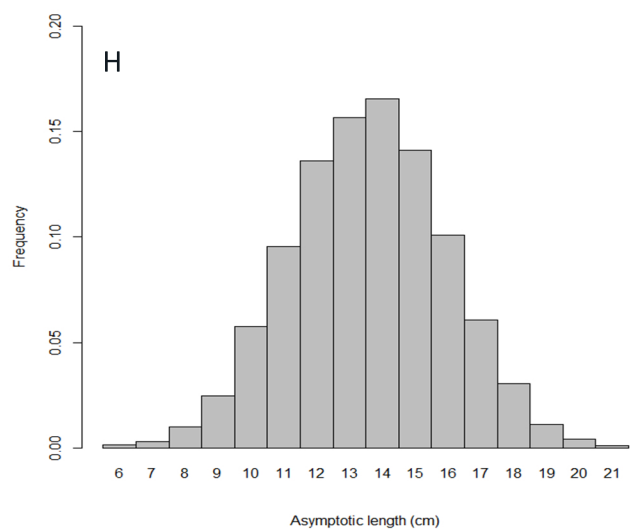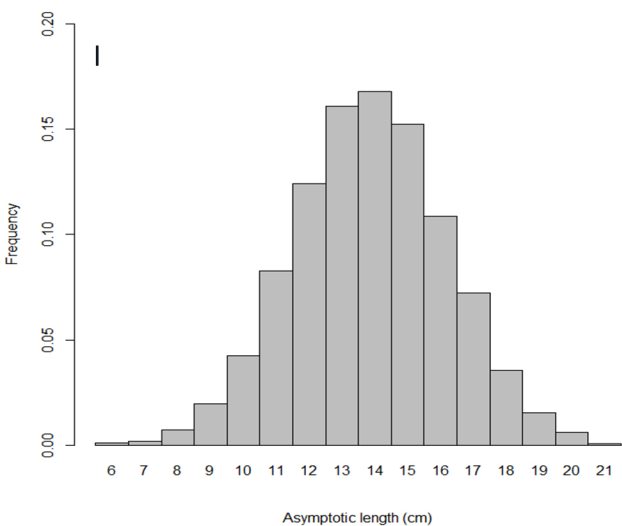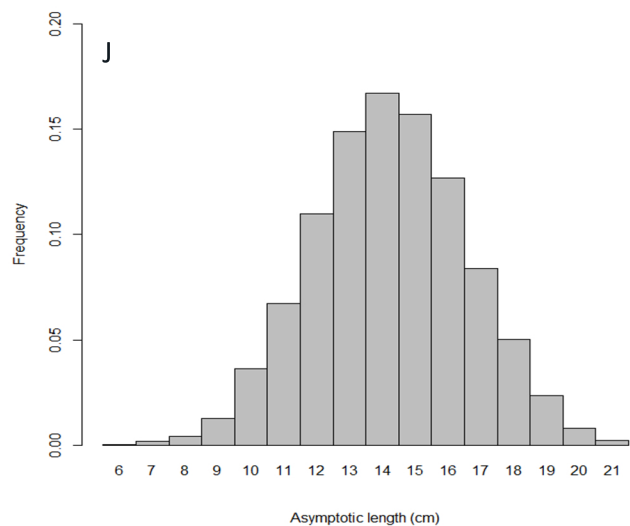

Supplement: Supplementary file 5 — Fig S5 [file ECE3-11-13363-s003.pdf]

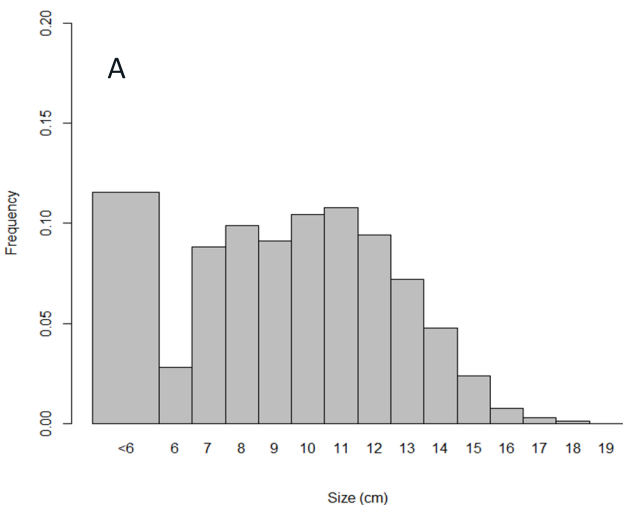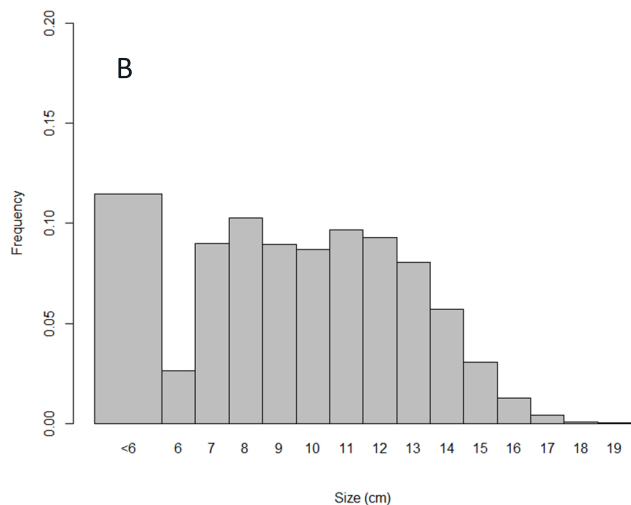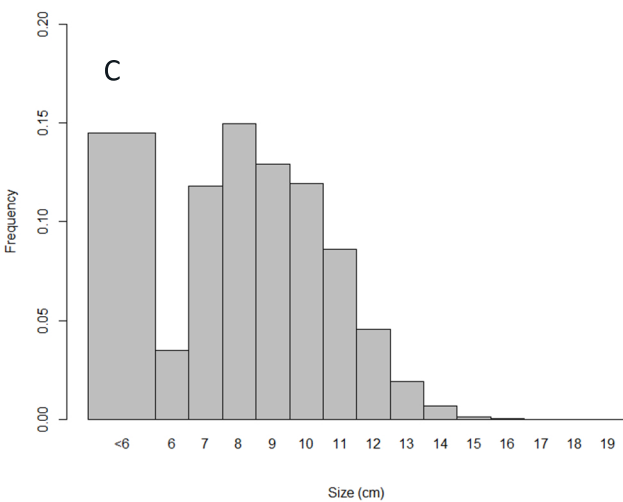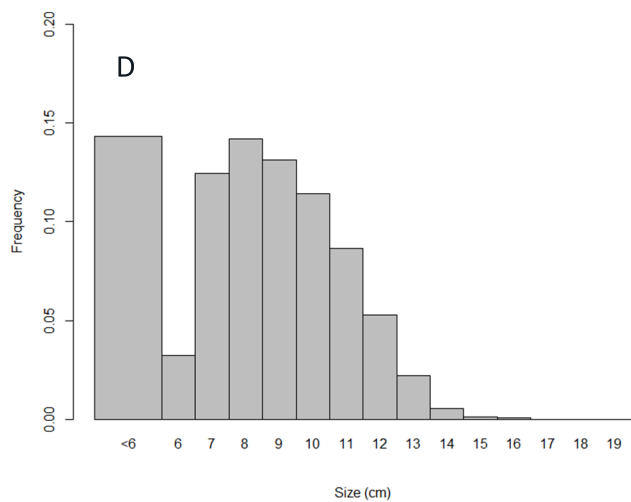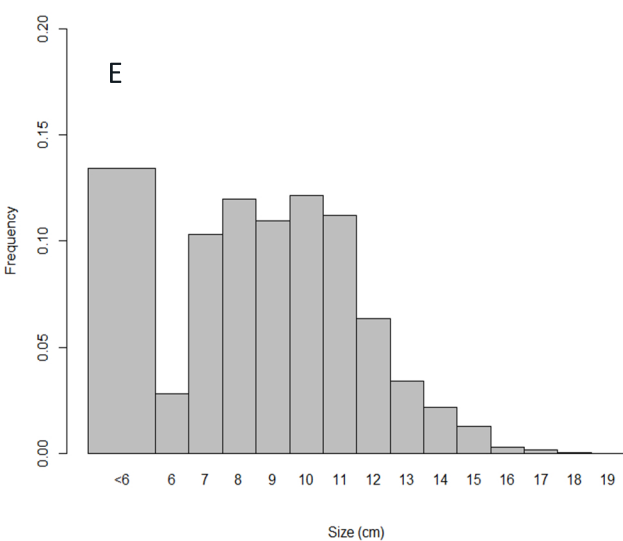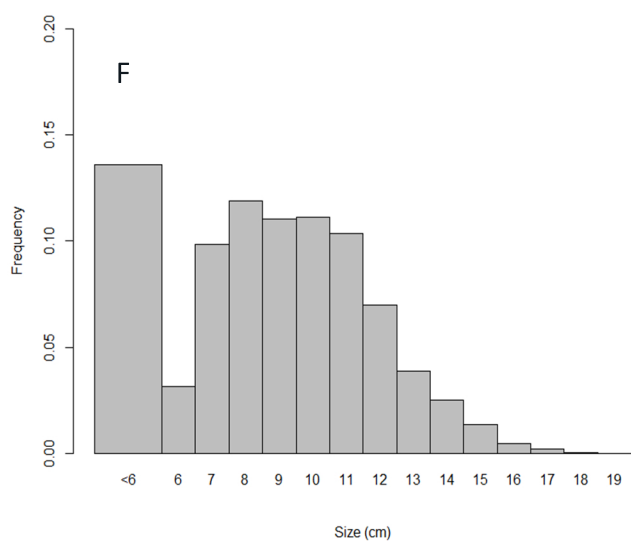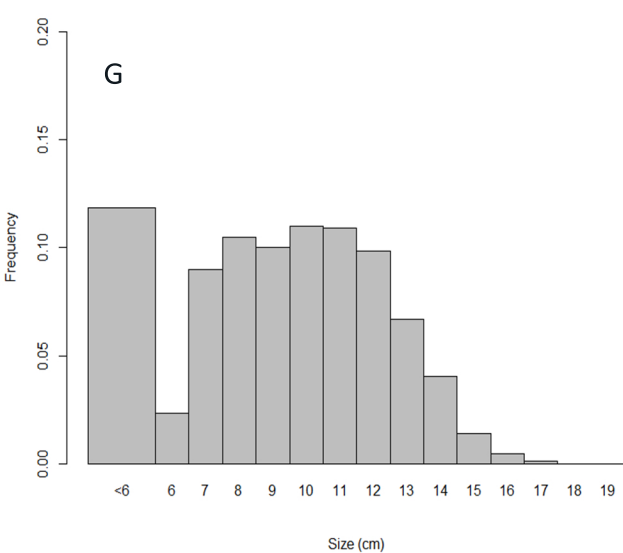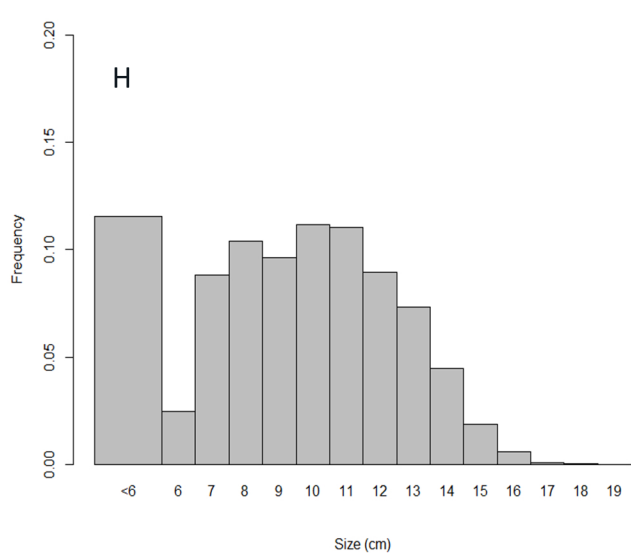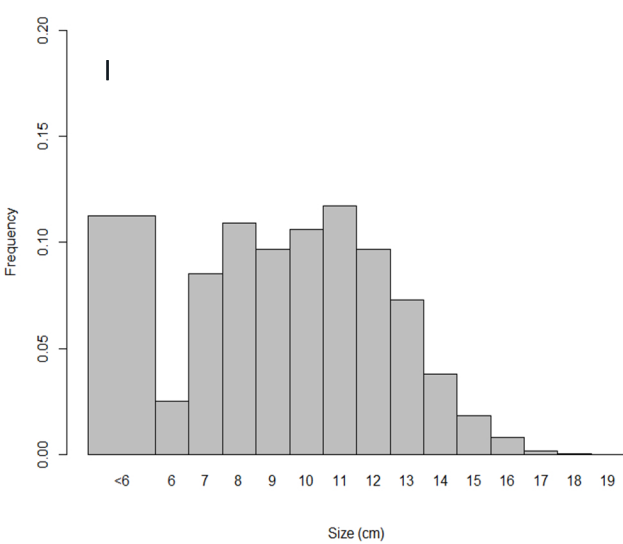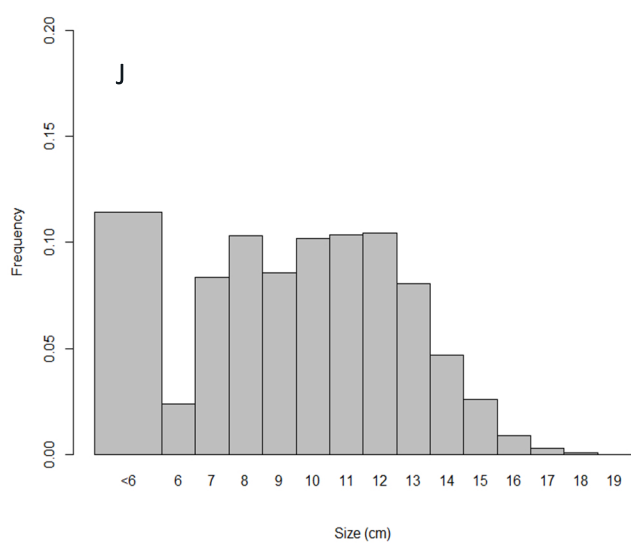

Supplement: Supplementary file 6 — Fig S6 [file ECE3-11-13363-s005.pdf]

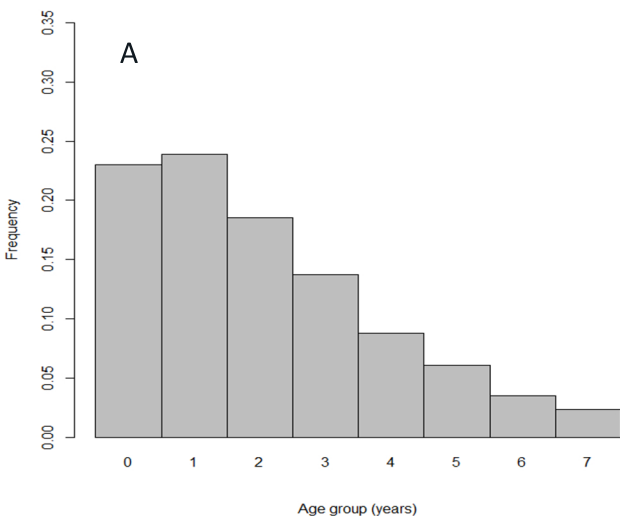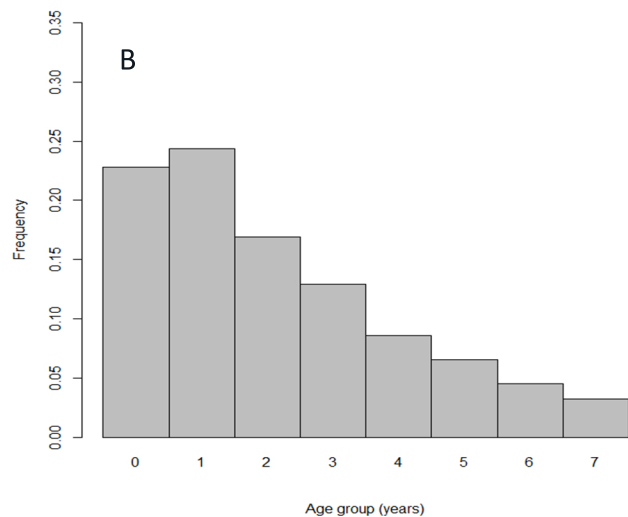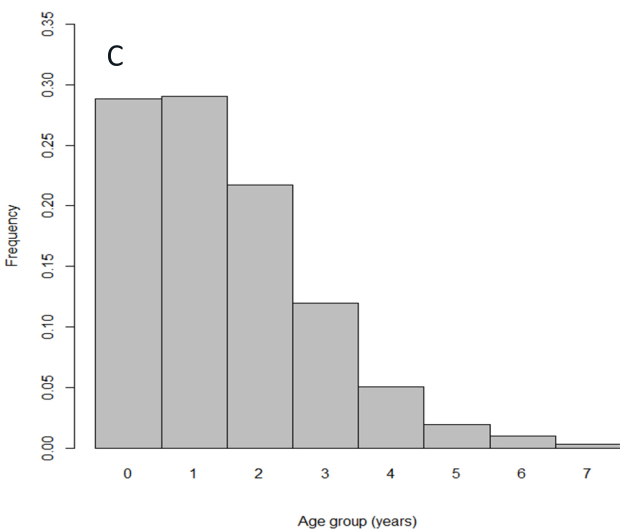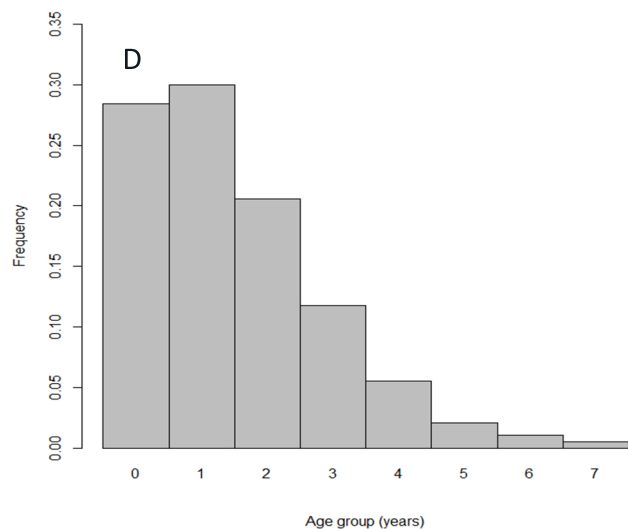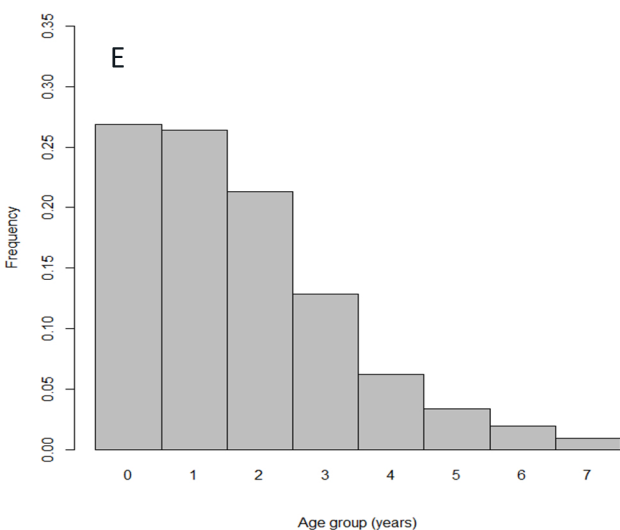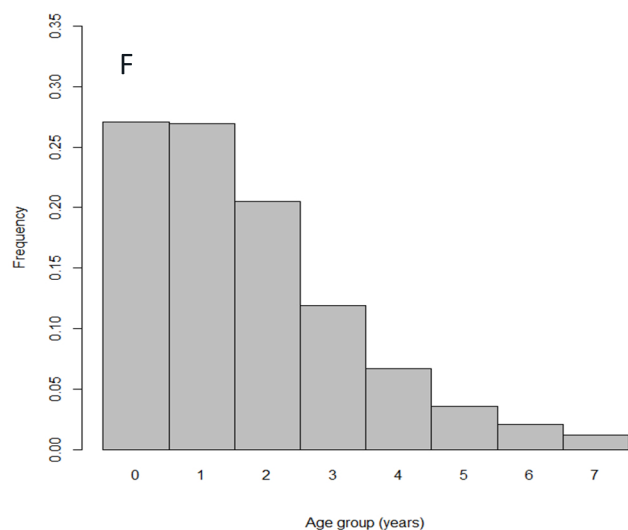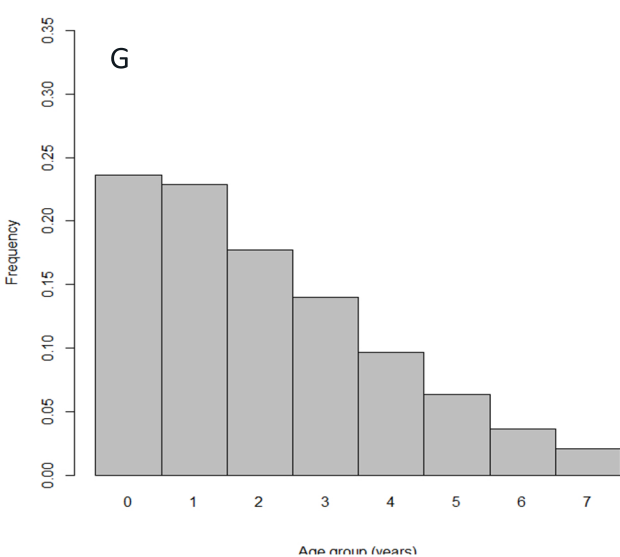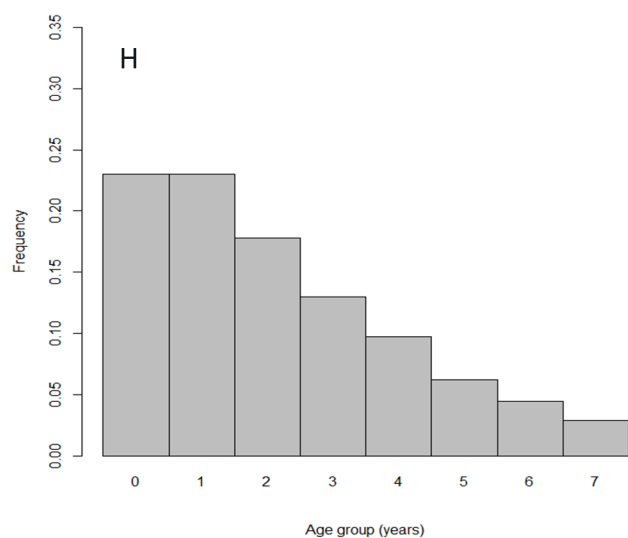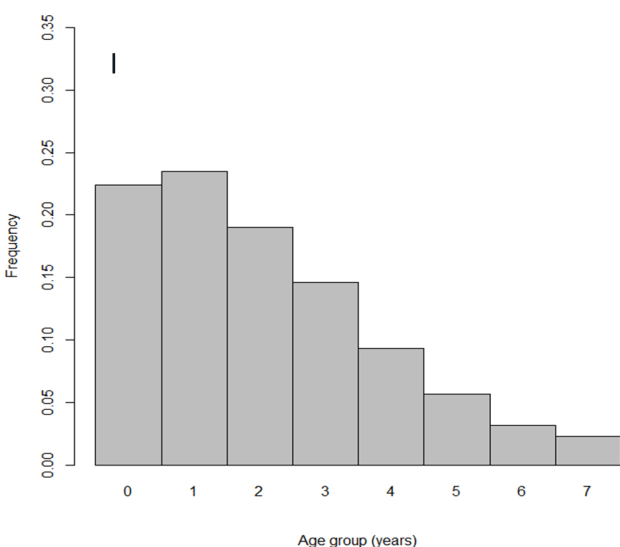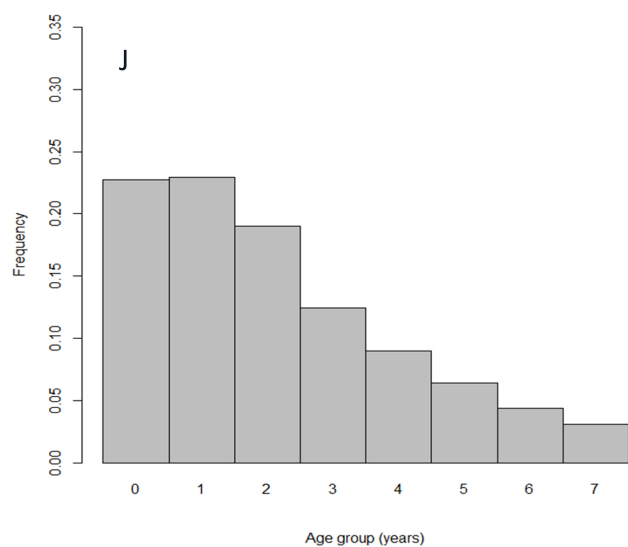

Supplement: Supplementary file 7 — Fig S7 [file ECE3-11-13363-s008.pdf]
