# Supplementary material for: Age is not just a number—Mathematical model suggests senescence affects how fish populations respond to different fishing regimes
Source: Ecol Evol. 2021 Sep 7;11(19):13363–78. doi: 10.1002/ece3.8058 (PMC8495815; doi:10.1002/ece3.8058)
Supplement: Supplementary file 8 — Appendix S1‐7‐Legend [file ECE3-11-13363-s004.docx]

**Appendix S1.** The lifetime cumulative number of offspring in each age group during the last ten years before fishing commences and the population is in equilibrium, and during the last ten years of intensive fishing (after 90 years of constant fishing). The red boxplots denote scenarios without senescence and black and white boxplots denote scenarios with both actuarial and reproductive senescence present.

**Appendix S2.** The lifetime cumulative number of offspring in each age group during the last ten years before fishing commences and the population is in equilibrium, and during the last ten years of intensive fishing (after 90 years of constant fishing). The green boxplots denote scenarios with reproductive senescence and blue boxplots denote scenarios with actuarial senescence present.

**Appendix S3.** Variation in asymptotic length (cm) between different simulation runs for the senescent scenario. Panels **a – j** represent individual, random simulations, one simulation per panel.

**Appendix S4.** Variation in asymptotic length (cm) between different simulation runs for the non-senescent scenario. Panels **a – j** represent individual, random simulations, one simulation per panel.

**Appendix S5.** The histogram shows the frequency of evolved asymptotic lengths (in cm). X-axis shows the length in cm so that 6 denotes lengths between 6cm and <7cm, 7 denotes lengths between 7cm and < 8cm, … and 21 denotes lengths between 21 cm and < 22 cm. Because different scenarios and phases (i.e. before, during and after fishing) had different numbers of fish, we present the frequency of each asymptotic length group instead of the absolute number of fish in each asymptotic length group, for ease of comparison. The different panels are as follows: **a)** Senescence scenario before fishing, **b)** No senescence scenario before fishing, **c)** Senescence scenario during fishing (logistic selection), **d)** No senescence scenario during fishing (logistic selection), **e)** Senescence scenario during fishing (dome-shaped selection), **f)** No senescence scenario during fishing (dome-shaped selection), **g)** Senescence scenario after fishing (logistic selection), **h)** No senescence scenario after fishing (logistic selection), **i)** Senescence scenario after fishing (dome-shaped selection), **j)** No senescence scenario after fishing (dome-shaped selection).

**Appendix S6.** The size (cm) distribution of fish. The different panels are as follows: **a)** Senescence scenario before fishing, **b)** No senescence scenario before fishing, **c)** Senescence scenario during fishing (logistic selection), **d)** No senescence scenario during fishing (logistic selection), **e)** Senescence scenario during fishing (dome-shaped selection), **f)** No senescence scenario during fishing (dome-shaped selection), **g)** Senescence scenario after fishing (logistic selection), **h)** No senescence scenario after fishing (logistic selection), **i)** Senescence scenario after fishing (dome-shaped selection), **j)** No senescence scenario after fishing (dome-shaped selection).

**Appendix S7.** The age distribution of fish. The different panels are as follows: **a)** Senescence scenario before fishing, **b)** No senescence scenario before fishing, **c)** Senescence scenario during fishing (logistic selection), **d)** No senescence scenario during fishing (logistic selection), **e)** Senescence scenario during fishing (dome-shaped selection), **f)** No senescence scenario during fishing (dome-shaped selection), **g)** Senescence scenario after fishing (logistic selection), **h)** No senescence scenario after fishing (logistic selection), **i)** Senescence scenario after fishing (dome-shaped selection), **j)** No senescence scenario after fishing (dome-shaped selection).
